# Recurrent somatic mutations and low germline predisposition mutations in Korean ALL patients

**DOI:** 10.1038/s41598-021-88449-4

**Published:** 2021-04-26

**Authors:** Sang-Yong Shin, Hyeonah Lee, Seung-Tae Lee, Jong Rak Choi, Chul Won Jung, Hong Hoe Koo, Sun-Hee Kim

**Affiliations:** 1 Department of Laboratory Medicine, Mokpo Jung Ang Hospital, Mokpo, Korea; 2 Brain Korea 21 PLUS Project for Medical Science, Yonsei University, Seoul, Korea; 3 Department of Laboratory Medicine, Yonsei University College of Medicine, 50 Yonsei-ro, Seodaemun-gu, Seoul, 03722 Korea; 4 Division of Hematology-Oncology, Department of Medicine, Samsung Medical Center, Sungkyunkwan University School of Medicine, Seoul, Korea; 5 Department of Pediatrics, Samsung Medical Center, Sungkyunkwan University School of Medicine, Seoul, Korea; 6 Department of Laboratory Medicine and Genetics, Samsung Medical Center, Sungkyunkwan University School of Medicine, 81, Irwon-ro, Gangnam-gu, Seoul, 06351 Korea

**Keywords:** Biological techniques, Cancer, Genetics, Molecular biology, Biomarkers, Diseases, Molecular medicine, Oncology, Pathogenesis

## Abstract

In addition to somatic mutations, germline genetic predisposition to hematologic malignancies is currently emerging as an area attracting high research interest. In this study, we investigated genetic alterations in Korean acute lymphoblastic leukemia/lymphoma (ALL) patients using targeted gene panel sequencing. To this end, a gene panel consisting of 81 genes that are known to be associated with 23 predisposition syndromes was investigated. In addition to sequence variants, gene-level copy number variations (CNVs) were investigated as well. We identified 197 somatic sequence variants and 223 somatic CNVs. The *IKZF1* alteration was found to have an adverse effect on overall survival (OS) and relapse-free survival (RFS) in childhood ALL. We found recurrent somatic alterations in Korean ALL patients similar to previous studies on both prevalence and prognostic impact. Six patients were found to be carriers of variants in six genes associated with primary immunodeficiency disorder (PID). Of the 81 genes associated with 23 predisposition syndromes, this study found only one predisposition germline mutation (*TP53*) (1.1%). Altogether, our study demonstrated a low probability of germline mutation predisposition to ALL in Korean ALL patients.

## Introduction

B-cell precursor and T-cell precursor acute lymphoblastic leukemia/lymphoma (B-ALL, T-ALL) are two of the most common malignancies in children. ALL can be classified by genetic alterations, which are highly various and heterogeneous. Chromosome aneuploidy, structural alterations and rearrangements, copy number variations (CNVs), and sequence mutations all contribute to leukemogenesis. In 2016, the fourth edition of the World Health Organization (WHO) classification of lymphoid and myeloid neoplasms and acute leukemia included new provision entities of ALL: *BCR-ABL1*-like (or Ph-like) ALL, *iAMP21* (intrachromosomal amplification of chromosome 21), and early T-cell precursor ALL (ETP-ALL)^1^.

*BCR-ABL1*-like ALL is a high-risk form of ALL with its peak incidence in young adults. *IKZF1* deletions, mutations of JAK-STAT and RAS signaling genes (*NRAS*, *KRAS*, *PTPN11*, *NF1,* etc.), and structural rearrangements (*CRLF2*, *ABL*-class tyrosine kinase genes, *JAK2*, *EPOR*, etc.) have been identified in this group^2^. *iAMP21* accounts for about 2% of ALL in older children, and it is associated with a number of adverse outcomes^3^. In *iAMP21*, three or more additional copies of *RUNX1* (*AML1*) are observed on chromosome 21 in metaphase fluorescence in situ hybridization (FISH). ETP-ALL is defined as CD1a^−^, CD8^−^, CD5^−^ (dim), and positive for one or more stem-cell or myeloid antigens. Genetic alterations in ETP are somewhat different than those in non-ETP, and *FLT3*, *DNMT3A*, and *WT1* mutations are more often found in ETP than in non-ETP^4, 5^.

In addition to somatic mutations, germline genetic predisposition to hematologic malignancies has emerged as an area of research interest. Genes found to be associated with predisposition to myeloid malignancy have been included in the WHO classification, “Myeloid neoplasms with germ line predisposition”^1^; this category includes *CEBPA, DDX41, RUNX1, ANKRD26, ETV6, GATA2,* and others. A number of syndromes, such as bone marrow failure syndrome and telomere biology disorders, are also included in that category. One early study estimated that childhood leukemia with hereditary genetic causes accounted for 2.6% of all cancers^6^. Down syndrome (DS) is the most common underlying genetic predisposition for ALL^6, 7^. There are a number of other syndromes that also increase susceptibility to ALL, such as Li Fraumeni (*TP53*), Bloom syndrome (*BLM*), Wiskott Aldrich syndrome (*WAS*), ataxia telangiectasia (*ATM*), and Nijmegen breakage syndrome (*NBN*)^8^. A germline mutation of *PAX5* is highly susceptible to the development of B-ALL^9^.

Some genes associated with ALL are found to have both germline and somatic mutations. For example, *PAX5*, *ETV6*, *TP53*, and *IKZF1* are known to have important somatic alterations, and germline mutations of those genes also cause susceptibility to ALL. Further, somatic and germline mutations of those genes can be found at the same time in leukemic samples^9, 10^. Therefore, upon initial ALL diagnosis, discrimination between somatic and germline mutations is a crucial aspect of accurately classifying ALL patient genetic subtypes/risk groups and detecting predisposition genes.

In this study, we used extensive gene panel sequencing to investigate genetic alterations (both somatic and germline) in Korean ALL patients; we also evaluated the clinical significance of recurrent somatic mutations and germline predisposition mutations in Korean ALL patients.

## Results

### Patients

Table 1 summarizes the characteristics of all 93 enrolled Korean ALL patients that are examined in this study. We enrolled 65 pediatric (< 20 years old) ALL patients and 28 adult ALL patients; this population included 12 T-ALL patients (seven children, five adults).

**Table 1:** Clinical characteristics of 93 ALL patients.

	Childhood	Adult
**Sex**		
Male	35	10
Female	30	18
**Diagnosis**		
B-ALL, NOS	25	8
B-ALL with t(9;22)(q34.1;q11.2); *BCR-ABL1*	2	11
B-ALL with t(v;11q23.3); *KMT2A* rearranged	4	1
B-ALL with t(12;21)(p13.2;q22.1); *ETV6-RUNX1*	9	
B-ALL with hyperdiploidy	16	1
B-ALL with t(1;19)(q23;p13.3); *TCF3-PBX1*	2	2
T-ALL	5	5
Early T-cell precursor acute leukemia	2	
**Cytogenetic risk group (B-ALL)**		
Good	26	2
Intermediate	26	9
High	6	12
	65	28

(1) good risk—*ETV6-RUNX1* and high hyperdiploidy (51–65 chromosomes); (2) intermediate risk—*TCF3-PBX1*, IGH translocations, B-other (none of these established abnormalities); (3) high risk—*BCR-ABL1, KMT2A* translocations, near haploidy (30–39 chromosomes), low hypodiploidy (less than 30 chromosomes), iAMP21, *TCF3-HLF.*

B-ALL with *BCR-ABL1* was the most common form of adult B-ALL (11/23). B-ALL, hyperdiploidy and B-ALL, and NOS were the most common forms of childhood B-ALL (41/58). B-ALL with t(12;21)(p13.2;q22.1); *ETV6-RUNX1* and B-ALL with t(v;11q23.3); *KMT2A* rearrangement respectively occurred in nine patients and four patients. The immunophenotyping results indicate that two adults with T-ALL were diagnosed with early T-cell precursor acute leukemia.

B-ALL was divided into three risk groups based on the classifications presented in a previous study^11^: (1) Good risk; *ETV6-RUNX1* and high hyperdiploidy (51–65 chromosomes); (2) Intermediate risk; *TCF3-PBX1*, *IGH* translocations and B-other (none of these established abnormalities); and (3) High risk; *BCR-ABL1*, *KMT2A* translocations, near haploidy (30–39 chromosomes), low hypodiploidy (less than 30 chromosomes), iAMP21, and *TCF3-HLF*.

### Germline sequence variants among 23 syndrome-associated genes

#### Pathogenic or likely pathogenic variants

Only one *TP53* variant was identified (Table 2). The *TP53* NM_000546.5: c.733G > A variant was identified in a B-ALL, NOS patient. The *TP53* NM_000546.5: c.733G > A variant has previously been reported in Li-Fraumeni syndrome patients (multiple cancers, including breast cancer, liver cancer, and lung cancer)^12^.

**Table 2:** Germline pathogenic/likely pathogenic variants identified in Korean ALL patients.

Patients	Sex/age	Diagnosis	Gene	Accession	Nucleotide	Amino acid	%Variant	dbSNP	Syndrome	Inheritance
ALL0009	Female/51	B-ALL, NOS	*TP53*	NM_000546.5	c.733G > A	p.Gly245Ser	40.3	rs28934575	Li-Fraumeni syndrome	Autosomal dominant
ALL0067	Male/18	B-ALL, NOS	CASP10*	NM_032977.3	–	–	–	–	Autoimmune lymphoproliferative syndrome, type II	Autosomal dominant

*Exonic deletion, exon 6-exon 9.

#### Germline copy number variants

Only one patient (male, 18 years old, B-ALL-NOS) had a known CNV, *CASP10* (deletion of exon 6-exon 9 which contained the CASc domain, as shown in Supplemental Fig. S1, Table 2). This same CNV was previously found in a patient with systemic juvenile idiopathic arthritis with incomplete penetrance^13^. *CASP10* is a causative gene for autoimmune lymphoproliferative syndrome (ALPS) type IIa, and its mutation hot spot is the protease domain (CASc) with missense mutation. However, our patient with the *CASP10* CNV has no clinical symptoms consistent with ALPS.

#### PID-associated germline sequence variants

Five PID-associated gene variants were identified in five patients (Supplement Table S1). All these variants were heterozygous autosomal recessive (AR) PID associated variants. Three variants (*IL12RB1, CTC1,* and *LPIN2*) have yet to be published, while the other variants (*TYK2* and *LIG4*) were known variants.

#### Overall somatic alteration of B-ALL and T-ALL

**Figure 1:**
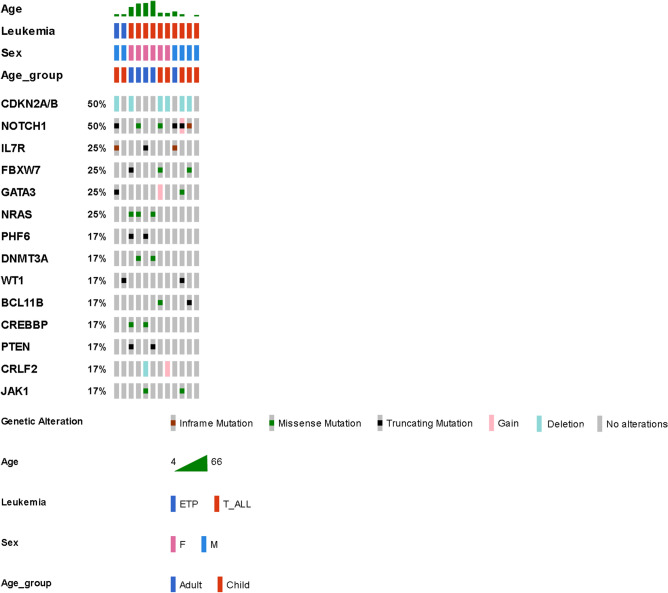
Most common genetic alterations in T-cell lymphoblastic leukemia/lymphoma (T-ALL) and ETP (Early T-cell Precursor Acute Leukemia). Only ≥ two patients with gene-alterations are shown in the figure. Data were analyzed by OncoPrinter (cBioPortal Version 1.14.0, Gao et al., Sci. Signal. 2013 and Cerami et al., Cancer Discov. 2012). Truncating mutations (nonsense, frameshift deletion, frameshift insertion, splice site); inframe (inframe deletion, inframe insertion).

**Figure 2:**
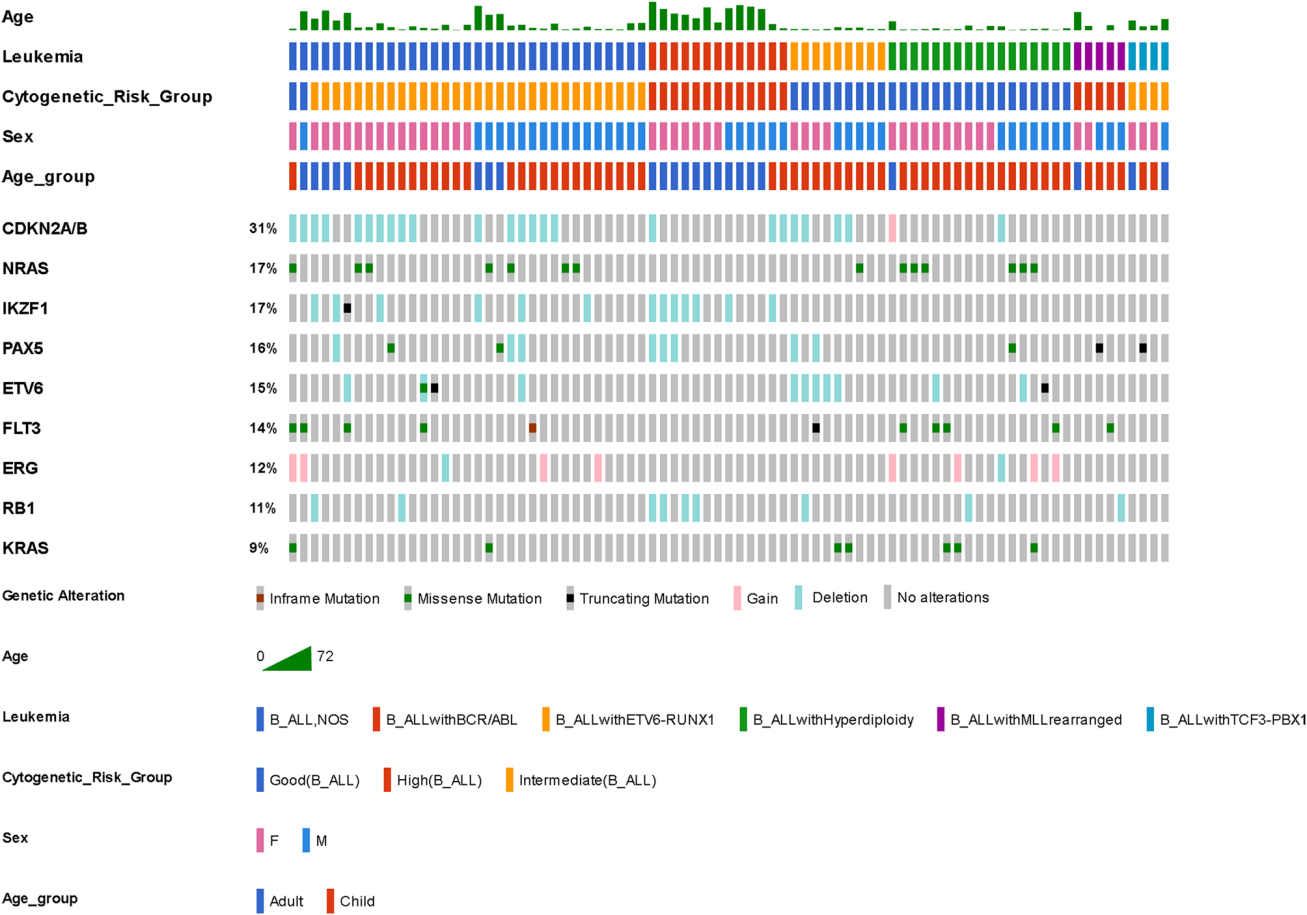
Most common genetic alterations in B-cell lymphoblastic leukemia/lymphoma (B-ALL). Only ≥ seven patients with gene-alterations are shown in the figure. Data were analyzed by OncoPrinter (cBioPortal Version 1.14.0, Gao et al., Sci. Signal. 2013 and Cerami et al., Cancer Discov. 2012). Truncating mutations (nonsense, frameshift deletion, frameshift insertion, splice site); inframe (inframe deletion, inframe insertion).

The most common genetic alterations are shown in Fig. 1 (T-ALL) and Fig. 2 (B-ALL). The most common genetic alterations of T-ALL were *NOTCH1* (50%), *CDKN2A/B* (50%), *IL7R* (25%), *FBXW7* (25%), *GATA3* (25%), and *NRAS* (25%). The most common genetic lesions in T-ALL were *NOTCH* (58%), Chromatin structure modifiers and epigenetic regulators (58%), and the cell cycle/*p53* signaling pathway (58%) (Supplement Fig. S2).

The most common (> 10%) genetic alterations of B-ALL were *CDKN2A/B* (31%), *NRAS* (17%), *IKZF1* (17%), *PAX5* (16%), *ETV6* (15%), *FLT3* (14%), *ERG* (12%), and *RB1* (11%). The most common genetic lesions in B-ALL were symphoid development and differentiation (49%), the cell cycle/*p53* signaling pathway (42%), and the RAS pathway (36%) (Supplement Fig. S3).

### Somatic sequence variants

We identified 197 variants after excluding synonymous variants (Supplement Table S2). *NOTCH1* (50%), *FBXW7* (25%), *IL7R* (25%), *NRAS* (25%), *DNMT3A* (17%), *PHF6* (17%), and *GATA3* (17%) were the most common sequence variants in B-ALL (Supplement Fig. S5). Meanwhile, *NRAS* (17%), *FLT3* (14%), *KRAS* (9%), *SETD2* (7%), *PAX5* (6%), and *CREBBP* (6%) were the most common sequence variants in B-ALL (Supplement Fig. S5).

Lollipop plots of the variants are shown in Supplemental Fig. S6. All *NRAS* variants were previously reported variants (COSMIC database, Supplement Table S2) or the same codon variant (p.Gly12Asp/Ser, p.Gly13Asp/Val, p.Gln61Arg/Lys). All *KRAS* variants were also previously reported variants or the same codon variant (p.Gly12Asp/Ser, p.Gly13Asp, p.Leu23Arg, p.Ala146Val/Thr). Most of the *FLT3* variants were known variants (previously found in hematologic malignancies, p.Asp835Asn/Tyr, p.Leu576Gln, p.Asn676Lys, et al.). About 50% of the *PAX5* variants were also recurrent variants (p.Val26Gly, p.Ala322ArgfsTer19, p.Val26Gly).

Five of the nine *NOTCH1* variants were known variants that had been found in hematologic malignancies (p.Arg1598Pro, p.Gln2503Ter, p.Glu2460Ter, p.Leu1585Gln, p.Phe1592Ser). Three of the four *FBXW7* variants were known variants that were found in hematologic malignancies (p.Arg465His, p.Arg479Gln, p.Arg465His). Most of *SETD2* were novel variants with no obvious hot spot mutations (seven of the eleven variants were frameshift or non-sense), as in the previous study^14^. The *CREBBP* variants were commonly found (4/8) in the histone acetyltransferase (HAT) domain with missense mutations, consistent with the results of a previous study^15^. Most of the *PTPN11*variants (4/5) were found in the Src homology 2 domain (SH2 domain, N-SH2 and C-SH), which are predominant region of mutation in hematologic diseases^16^.

### Somatic copy number variants

We found a total of 223 somatic CNV. *CDKN2A/B* (50%), *CRLF2* (17%), *GATA3* (8%), *CSF2RA* (8%), and *BCOR* (8%) were the most common CNV in T-ALL (Supplement Fig. S7). *CDKN2A/B* (31%), *IKZF1* (16%), *ETV6* (12%), *ERG* (12%), and *RB1* (11%) were the most common CNV in B-ALL (Supplement Fig. S8).

Fifteen B-ALL patients had an *IKZF1* alteration (Table 3). Seven patients had B-ALL with t(9;22)(q34;q11.2); *BCR-ABL1*. Six B-ALL, NOS patients had an *IKZF1* gene deletion. Seven of thirteen B-ALL with t(9;22)(q34;q11.2); *BCR-ABL1* patients had an *IKZF1* deletion (54%). Three of thirteen B-ALL with t(9;22)(q34;q11.2); *BCR-ABL1* patients had a *PAX5* deletion (23%).

**Table 3:** *IKZF1* alteration cases.

Patients	Sex	Age group	Diagnosis	Bone marrow transplant	*IKZF1*	*PAX5*	Relapse	Clinical outcome
ALL0003	Female	Adult	B-ALL with *BCR-ABL1*	–	Deletion	Deletion	–	–
ALL0004	Female	Adult	B-ALL with *BCR-ABL1*	–	Deletion	Deletion	–	Expired
ALL0010	Female	Adult	B-ALL with *BCR-ABL1*	Allogenic	Deletion	–	–	–
ALL0011	Male	Adult	B-ALL, NOS	–	Deletion	–	Yes	Expired
ALL0024	Female	Child	B-ALL, NOS	–	Deletion	–	Yes	–
ALL0026	Male	Child	B-ALL, NOS	Allogenic	Deletion	–	Yes	Expired
ALL0028	Female	Adult	B-ALL, NOS	Allogenic	Deletion	–	Yes	Expired
ALL0047	Male	Child	Early T-cell precursor acute leukemia	Allogenic	Missense mutation	–	Yes	Expired
ALL0049	Female	Adult	B-ALL, NOS	Allogenic	Deletion	Deletion	–	–
ALL0051	Male	Child	B-ALL, NOS	–	Deletion	Deletion	–	–
ALL0062	Male	Adult	B-ALL with *BCR-ABL1*	–	Deletion	–	–	–
ALL0064	Female	Adult	B-ALL with *BCR-ABL1*	Allogenic	Deletion	–	–	–
ALL0065	Female	Adult	B-ALL, NOS	Allogenic	Frameshift mutation	–	–	–
ALL0069	Male	Child	B-ALL with *BCR-ABL1*	Allogenic	Deletion	–	–	–
ALL0083	Female	Adult	B-ALL with *BCR-ABL1*	–	Deletion	Deletion	–	–

### *CDKN2A/B* by NGS and FISH

FISH for *CDKN2A/B* was performed upon the initial diagnosis of ALL. We compared the *CDKN2A/B* results between the FISH and NGS CNVs analyses. The overall agreement rate for *CDKN2A/B* was 83.7% (Table 4). Nine cases were positive for *CDKN2A/B* deletion according to NGS, but negative by FISH. Meanwhile, six cases were normal by NGS analysis, but deletion/duplication was confirmed by FISH.

**Table 4:** Comparison of FISH and NGS results for *CDKN2A/B* deletion/duplication.

NGS (next generation sequencing) results				
	Deletion	Duplication	Normal	Total
**FISH (fluorescent in situ hybridization) results**				
Deletion	20		1	21
Duplication		1	5	6
Normal	9		56	65
Not tested	1			1
Total	30	1	62	93

Agreement rate: 83.7%.

### Clinical effects of genetic alteration

#### Overall survival and relapse-free survival

**Figure 3:**
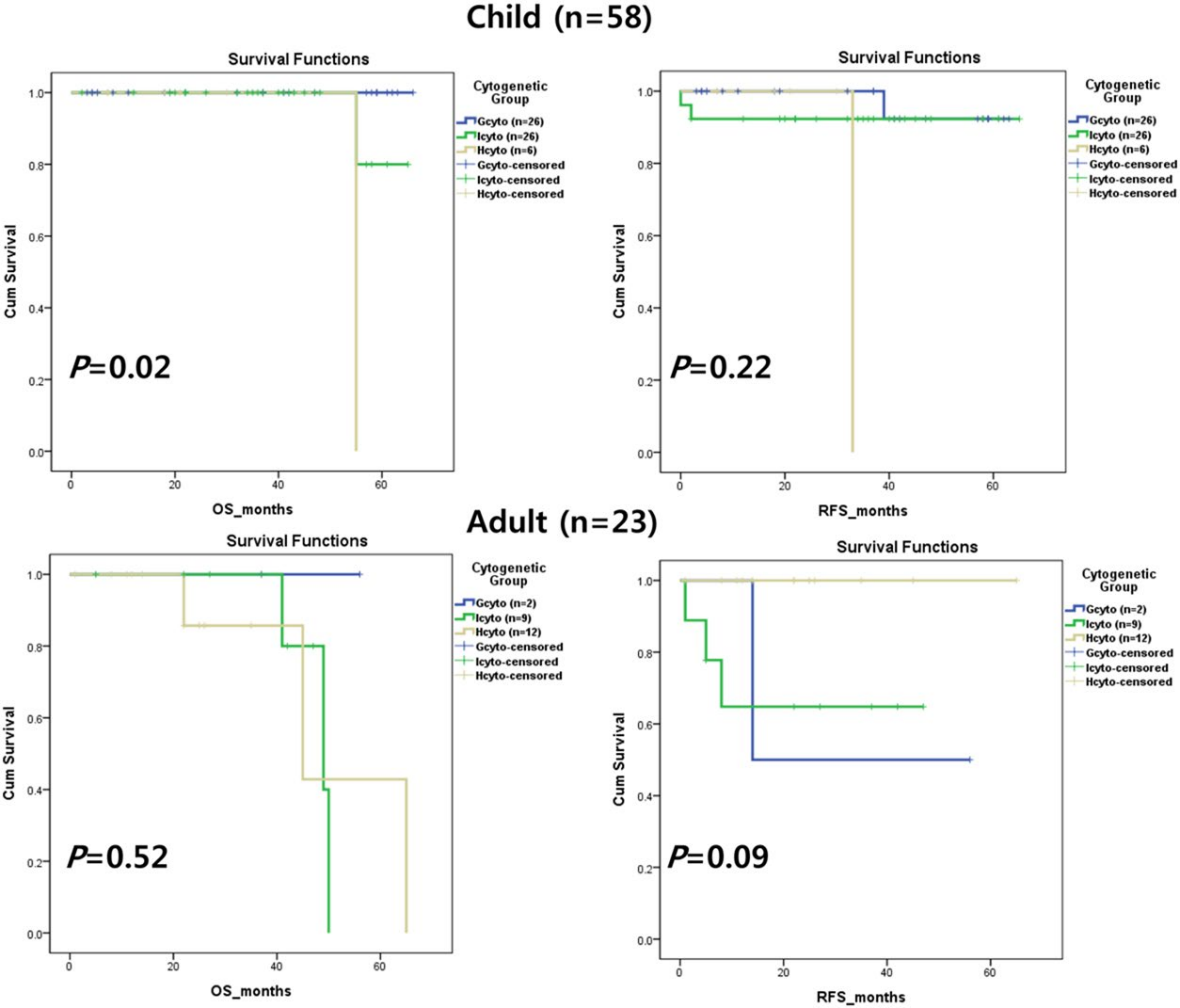
Clinical outcomes by cytogenetic group. Overall survival (OS) and relapse-free survival (RFS) were both found to be statistically significant (*P* < 0.5) in child B-cell lymphoblastic leukemia/lymphoma (B-ALL). RFS was statistically significant in adult B-ALL. Child B-ALL were classified into good cytogenetic (Gcyto, n = 26), intermediate cytogenetic (Icyto, n = 26), and high cytogenetic risk (Hcyto, n = 6) groups. Adult B-ALL were classified into Gcyto (n = 2), Icyto (n = 9), and Hcyto (n = 12) groups.

Overall survival (OS) and relapse-free survival (RFS) are shown by cytogenetic groups in Fig. 3. There were statistically significant differences in OS and RFS in childhood ALL, but not in adult ALL.

### Clinical impact of *IKZF1*

**Figure 4:**
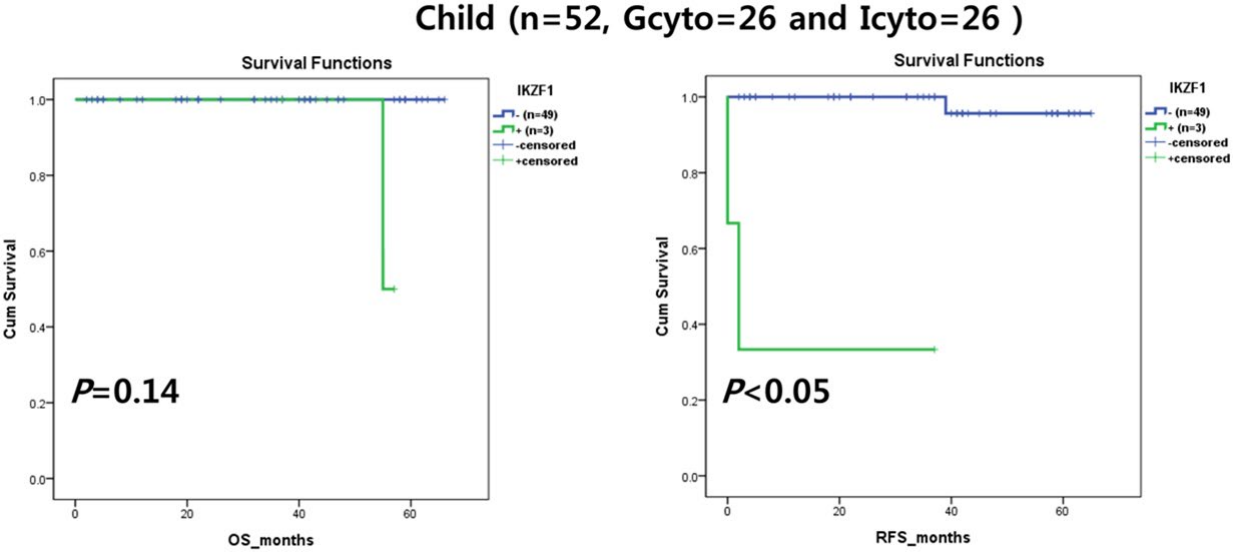
Clinical outcomes by *IKZF1* alteration. *IKZF1* alteration had a prognostic impact on overall survival (OS) and relapse-free survival (RFS) in child B-cell lymphoblastic leukemia/lymphoma (B-ALL), particularly in the good cytogenetic (Gcyto) and intermediate cytogenetic risk (Icyto) groups. Child B-ALL were classified into Gcyto (n = 26) and Icyto (n = 26) groups.

*IKZF1* alteration had adverse effects on OS and RFS only in childhood ALL (Fig. 4). No other genes had a consistent clinical effect in childhood or adult ALL (data not shown).

## Discussion

NGS technology has been applied to a number of hematologic diseases. Many gene panels and several methods have been used to detect not only sequence variants, but also large gene deletions and duplications or gene fusions^15–19^. In this study, we found a significant agreement rate between NGS and FISH for *CDKN2A/B* CNV detection. We also found significant *IKZF1* deletions using an NGS CNV analysis. A presumed diagnosis of *BCR-ABL1*-like ALL can be enabled using an NGS CNV analysis to test for genetic alterations in *IKZF1* and *JAK1/JAK2* because the former (68%) and latter (55% among patients with *CRFL2* rearrangement) are more prevalent in *BCR-ABL1*-like ALL than in other B-ALL sub-types^2^. Although we did not perform gene expression profiling and FISH, or RT-PCR for alterations commonly found in *BCR-ABL1*-like ALL, we found seven cases with *IKZF1* alterations in non-ALL with *BCR-ABL*, and these showed adverse clinical effects. We assume that the seven non-ALL patients with *BCR-ABL1* and an *IKZF1* alteration are likely *BCR-ABL1*-like ALL.

In B-ALL, the most common pathogenic pathways are RAS signaling (~ 48%, *NRAS*, *KRAS*, *PTPN11*, *FLT3*, *NF1, *etc.) and Lymphoid development/differentiation (18–80%, *PAX5*, *IKZF1*, *EBF1*, etc.)^2, 20, 21^, and we found a similar distribution of genetic changes in this study (42% RAS signaling and 49% Lymphoid development/differentiation variants were identified). The deletion of *IKZF1* (~ 41.4%), *CDKN2A/B* (~ 36.9%), and *PAX5* (~ 25.5%) are most common in overall B-ALL^21, 22^. *IKZF1* deletion has a poor prognostic effect and is more frequent (60–90%) in B-ALL with *BCR-ABL1* or high risk non-*BCR-ABL1* ALL (~ 30%) than other B-ALL subtype, as shown in our results^2, 11, 22, 23^. Consistent with previous studies, our study showed that *IKZF1* deletion was more frequent in the adult high-risk cytogenetic group (6/12, 50%) than in the overall B-ALL group (13/81, 16%). This high prevalence of *IKZF1* deletion in the adult high-risk cytogenetic group might have occurred because most cases (11/12) were ALL with *BCR-ABL1*.

In T-ALL, we found recurrent somatic sequence variants and CNVs similar to those reported in previous studies^4, 5^. The sequence variants of *NOTCH1* (~ 50%), *PHF6* (~ 20%), *JAK3-IL7R* (~ 30%), and *FBXW7* (~ 18%) are common mutations in T-ALL^4, 5, 22–24^. *CDKN2A/B* deletion (50%, 6/12) is most common, as shown in previous studies (50–70%)^22, 23^. Although only two cases of ETP were enrolled in this study, the identified variants were among the recurrent genes in T-ALL or ETP (*FLT3, WT1*, *NOTCH1*, and *IL7R*)^4, 5^. There is a need for more ETP cases to reveal the genetic alterations in T-ALL among Koreans.

Skin fibroblasts are the only recommended control sample for germline mutations, because peripheral blood (PB) and bone marrow can be contaminated with leukemic cells, while other samples, such as saliva or buccal swab, can be also contaminated with PB. Further, clonal hematopoiesis can be observed in ~ 10% of the healthy population, and this rate increases with age^25^. However, a skin biopsy is an invasive procedure, and as a result, such samples are not readily available. We therefore used CR-state bone marrow slides (acquired to test for residual leukemic cells) as the control for germline mutations. No apparent leukemic samples were obtained in our review of bone marrow morphology, or in the FISH, chromosome, flow-cytometry, and RT-PCR results. The variant allele fraction (VAF) and public population databases were used as filtering tools^26^. Germline variants can have a VAF of > 33%, even in tumor samples^26^; variants within that range have a high possibility of germline origin. Some presumably somatic variants registered in public databases, such as the Single Nucleotide Polymorphism database (dbSNP), the 1000 Genomes Project, the Exome Aggregation Consortium (ExAC) database, and the Human Gene Mutation Database (HGMD), may be of germline origin. We double checked the germline variants in both CR and leukemic samples. True germline variants (identified in CR samples) were also identified in paired leukemic samples with similar VAF. By contrast, true somatic variants (identified in leukemic samples) were either not found, or were found with very low VAF (< 1%) in paired CR.

Various syndromes increase the risk of ALL, with variable penetrance and preference. DS is the most common genetic cause of childhood leukemia. In an analysis of the National Registry of childhood tumors in the United Kingdom, 131 of 142 leukemia patients with underlying genetic causes were DS patients^6^, and an analysis of approximately 18,000 European childhood ALL cases found that 2.4% of ALL patients also had DS^7^. Other genetic diseases with connections to ALL include ataxia telangiectasia, Nijmegen breakage syndrome, neurofibromatosis type 1, familial ALL, and Noonan syndrome^7^. Germline *PAX5* and *ETV6* mutations carry a high risk (high penetrance) of cancer, mainly ALL^7, 9, 27^; bloom syndrome and constitutional mismatch repair deficiency syndrome also carry a moderate risk of ALL. Constitutional mismatch repair deficiency syndrome is more associated with T-cell lineage leukemia and lymphoma than B-cell lineage^28^. However, this study found no pathogenic or likely pathogenic variants among the gene mutations (*PAX5*, *ETV6*, *NF1*, *BLM*, *ATM*, etc.) that have high penetrance for ALL^7^. This may be because we enrolled a relatively small number of unselected sporadic cases. Moriyama et al. reported that only 0.79% (35/4,405) of sporadic childhood ALL cases have a potentially pathogenic *ETV6* variant^26^.

In this study, we did identify only one pathogenic variant (*TP53*) among 81 genes associated with 23 syndromes that are well known for their connection to hematologic malignancy. For example, Li-Fraumeni syndrome (*TP53*) is a well-known rare cancer syndrome. The most common cancers in patients with Li-Fraumeni syndrome are solid cancers (such as breast cancer, lung cancer, and bladder cancer)^12^. However, a somatic *TP53* alteration is strongly associated with low hypodiploidy ALL (~ 90%), disease relapse, and germline origin (~ 40%)^29^. Although hypodiploid ALL accounts for only 5% of childhood ALL cases, hypodiploid ALL patients should be tested for Li-Fraumeni syndrome because of its poor prognosis and the possibility of a germline *TP53* mutation^30^.

We found germline copy number variation in *CASP10*. *CASP10* is a causative gene for autoimmune lymphoproliferative syndrome (ALPS) type IIa, which is a very rare PID (primary immunodeficiency disorder). However, the association between exonic deletion of *CASP10* and leukemia in our patient is unclear in this study. In a previous study, similar exonic deletion of *CASP10* had only been found in a patient with systemic juvenile idiopathic arthritis with incomplete penetrance (healthy relative with the same CNV)^13^. In this case, we could not find any medical history associated with ALPS in our patient.

More than 300 distinct disorders and genes of PID have been classified by the International Union of Immunological Societies PID expert committee^31^. An increase in leukemia/lymphoma with PID (including ALPS) is well known and expected^32^. Although the mechanism of leukemogenesis in PID remains unclear, intrinsic (cancer predisposition parallel to the immunological defect) and extrinsic (chronic infections, inflammation, or loss of immunosurveillance) mechanisms have been proposed by Hauck et al.^33^. Notably, in this study, we identified five PID-associated sequence variants and one CNV. All these sequence variants were heterozygous autosomal recessive PID associated variants. Therefore, the association between these variants and leukemia in our patients is unclear. However, some studies have reported an increased risk of cancer in heterozygous carriers of autosomal recessive PID associated variants, heterozygous *BLM* (Bloom syndrome, AR) mutations, and heterozygous *ATM* (Ataxia-telangiectasia, AR) mutations^34, 35^. Moreover, Qin, N. et al. have reported a risk of subsequent cancer among long-term survivors of childhood cancer with germline pathogenic/likely pathogenic mutations (DNA repairs genes which are mostly included in PID-associated genes, such as *BLM*, *FANCA*, *BRCA2 LIG4*, *NBN*, etc.)^36^. Therefore, further studies should continue elucidating these uncertain significant variants of PID-associated variants.

Our study had several limitations. First, we enrolled a relatively small number of cases. Second, we did not use skin fibroblasts in our search for germline mutations. Third, we did not perform a familial study or any clinical or physical investigations of the identified germline variants.

## Conclusion

We found recurrent somatic alterations in Korean ALL patients. Further, we identified the low probability of germline mutation predisposition in unselected sporadic Korean ALL patients. We also demonstrated the usefulness of NGS technology, which provides comprehensive genetic information.

## Methods

### Study population and samples

We selected paired initial-diagnosis and complete remission (CR) bone marrow samples from patients diagnosed with ALL at Samsung Medical Center from 2008 to 2012.

The Institutional Review Board at Samsung Medical Center approved this study (IRB No. 2015-11-053), and informed consent was obtained from all participants. All experiments were performed in accordance with the relevant guidelines and regulations.

To detect germline mutations, we used bone marrow slides that were obtained when the patients were in CR. In total, 31 (33.3%) patients received allogenic stem cell transplantation. CR status bone marrow slides before allogenic stem cell transplantation were used to accurately detect patient germline mutations. The morphology, chromosome, FISH, and immunophenotyping results were reviewed, and bone marrow slides with no apparent residual leukemic cells were selected as control samples.

### Conventional study

A chromosome study was conducted using a standard method, and the karyotypes are described according to the International System for Human Cytogenetic Nomenclature. Multiplex reverse transcription polymerase chain reaction (RT-PCR) was performed to detect recurrent translocation (HemaVision kit, DNA Technology, Aarhus, Denmark). *FLT*-ITD mutation analyses (by fragment length polymorphism) and FISH for *CDKN2A/B* were performed as well.

### Targeted gene sequencing

#### Gene panel

From a literature review, we selected 500 genes found to be significantly mutated in ALL (Supplement Table S5). Our gene panel^17^ included the following: cell cycle and p53 signaling pathway (*ATM, CDKN1B, CDKN2A, CDKN2B, RB, TP53,* etc.), chromatin structure modifiers and epigenetic regulators (*ARID1A, BMI1, CHD1, CHD4, CHD9, CREBBP, CTCF, DNMT3A, EED, EP300, EZH2, KDM5C, KDM6A, KMT2A, KMT2C, KMT2D, NR3C1, PHF6, SETD2, SUZ12*, *WHSC1,* etc.), JAK-STAT signaling pathway (*CRLF2, IL2RB, IL7R, JAK1, JAK2, JAK3, PTPN2, SH2B3, STAT3*, *TYK2,* etc.), DNA repair (*MSH2, MSH6*, *ZFHX4, *etc.), NOTCH pathway (*FBXW7*, *NOTCH1,* etc.), PI3K-AKT-mTOR signaling pathway (*AKT2, PIK3CD, PIK3R1*, *PTEN,* etc.), RAS pathway (*BRAF, CBL, FLT3, KRAS, NF1, NRAS*, *PTPN11,* etc.), and transcriptional processes (*BCL11B, DNM2, ERG, GATA3, LMO2, MYB, RELN, TAL1, TBL1XR1, TLX1, TLX3*, *WT1,* etc.).

### Predisposition syndrome to hematologic malignancies

We included 23 well known predisposition syndromes (81 genes) in our next generation sequencing (NGS) panel: ataxia pancytopenia syndrome (*SAMD9L*), ataxia telangiectasia (*ATM*), Bloom syndrome (*BLM*), constitutional mismatch repair deficiency syndrome (*MLH1, MSH2, MSH6, PMS2*, and *EPCAM*), Diamond-Blackfan anemia (*GATA1, RPL5, RPL11, RPL15, RPL23, RPL26, RPL27, RPL31, RPL35a, RPL36, RPS7, RPS10, RPS15, RPS17, RPS19,RPS24, RPS26, RPS27, RPS27A, RPS28, RPS29*, and *TSR2*), dyskeratosis congenita (*DKC1, TERC, TERT, NOP10, NHP2, TINF2, WRAP53, CTC1, RTEL1, ACD, PARN*, and *NAF1*), familial acute myeloid leukemia (*CEBPa*), familial platelet disorder with propensity to myeloid malignancy (*RUNX1*), Fanconi anemia (*FANCA, FANCB, FANCC, BRCA2, FANCD2, FANCE, FANCF, FANCG, FANCI, BRIP1, FANCL, FANCM, PALB2, RAD51C, SLX4, ERCC4, RAD51, BRCA1,UBE2T, XRCC2*, and *MAD2L2*), GATA2-spectrum disorders (*GATA2*), Li Fraumeni (*TP53*), Ligase IV syndrome (*LIG4*), neurofibromatosis (*NF1*and *SRP72*), Nijmegen breakage syndrome (*NBN*), Noonan syndrome (*PTPN11*), Noonan-like syndrome (*CBL*), severe congenital neutropenia 3 (*DDX41* and *HAX1*), severe congenital neutropenia (*ELANE*), Shwachman-Diamond syndrome (*SBDS*), susceptibility to ALL 3 (*PAX5*), thrombocytopenia 2 (*ANKRD26*), thrombocytopenia 5 (*ETV6*), and Wiskott Aldrich Syndrome (*WAS*).

### Data analysis

The data analysis was conducted using previously described methods (Supplement Fig. S9)^17^. After sequencing, we aligned the reads to human genomic reference sequences (GRCh37) using the Burrows–Wheeler alignment tool. The Genome Analysis Tool Kit (Broad Institute) was used for variant calling. Pindel was used for crosscheck insertion and mutation deletion. All mutations were annotated using ANNOVAR and VEP software. Variants were further examined by visual inspection using the Integrative Genomic Viewer. Annotated variants were classified using automated algorithm software, DxSeq Analyzer (Dxome, Seoul, Korea), by applying the standards and guidelines of the American College of Medical Genetics and Genomics and the Association for Molecular Pathology^37^. ExomeDepth (1.1.10), an R package, was used to detect exon- and gene-level CNVs in target regions, followed by visualization using a base-level read depth normalization algorithm implemented in a DxSeq Analyzer (Dxome, Seoul, Korea)^17^. To obtain reliable results, we used cutoff values for the average depth and % covered (30×) of 700× and 99%, respectively. The minimal reportable VAF was ≥ 1%.

### Statistical analysis

Differences in survival according to mutation group were analyzed using Kaplan–Meier estimates. A *P*-value of < 0.05 was considered to be statistically significant. All statistical analyses were performed in PASW Statistics 20.0.

### Ethics declarations

The Institutional Review Board at Samsung Medical Center approved this study (IRB No. 2015-11-053), and informed consent was obtained from all participants.

## Supplementary Information

Supplementary Information.
