# Supplementary material for: Recurrent somatic mutations and low germline predisposition mutations in Korean ALL patients
Source: Sci Rep. 2021 Apr 26;11:8893. doi: 10.1038/s41598-021-88449-4 (PMC8076247; doi:10.1038/s41598-021-88449-4)
Supplement: Supplementary file 1 — Supplementary Information. [file 41598_2021_88449_MOESM1_ESM.docx]

**Recurrent Somatic Mutations and Low Germline Predisposition Mutations in Korean ALL patients**

Sang-Yong Shin^1^, Hyeonah Lee^2^, Seung-Tae Lee^3*^, Jong Rak Choi^3^, Chul Won Jung^4^, Hong Hoe Koo^5^ and Sun-Hee Kim^6*^

^1^Department of Laboratory Medicine, Mokpo Jung Ang Hospital, Mokpo, Korea

^2^Brain Korea 21 PLUS Project for Medical Science, Yonsei University, Seoul, Korea

^3^Department of Laboratory Medicine, Yonsei University College of Medicine, Seoul, Korea

^4^Department of Internal Medicine, Samsung Medical Center, Sungkyunkwan University School of Medicine, Seoul, Korea

^5^Department of Pediatrics, Samsung Medical Center, Sungkyunkwan University School of Medicine, Seoul, Korea

^6^Department of Laboratory Medicine and Genetics, Samsung Medical Center, Sungkyunkwan University School of Medicine, Seoul, Korea

*Correspondence to Sun-Hee Kim and Seung-Tae Lee

**Sun-Hee Kim, M.D., Ph.D.**

Department of Laboratory Medicine and Genetics, Samsung Medical Center, Sungkyunkwan University School of Medicine, 81, Irwon-ro, Gangnam-gu, Seoul, 06351, Korea.

Tel: +82-2-3410-2704. Fax: +82-2-3410-2719

E-mail: drsunnyhk@gmail.com

**Seung-Tae Lee, M.D., Ph.D.**

Department of Laboratory Medicine, Yonsei University College of Medicine, 50 Yonsei-ro, Seodaemun-gu, Seoul 03722, Korea.

Tel: +82-2-2228-2450. Fax: +82-2-364-1583

E-mail: LEE.ST@yuhs.ac

| **Supplement Table S1. PID-associated germline sequence variants identified in Korean ALL patients** | | | | | | | | | | | | |
| --- | --- | --- | --- | --- | --- | --- | --- | --- | --- | --- | --- | --- |
| **ID** | **Age/**  **Sex** | **Gene** | **Nucleotide/**  **Amino acid** | **%**  **Variant** | **dbSNP** | **GnomAD-exome** | **Global**  **(ExAC)** | **Genome**  **Asia** | **Korea1K** | **Korean**  **(KRGDB)** | **Diseases** | **PMID** |
| ALL0077 | 10/M | *TYK2* | c.209_212del/ p.Cys70SerfsTer21 | 40.2 | rs770927552 | 0.000048 | 0.00006591 | - | 0.0027 | - | Immunodeficiency 35, AR | 17088085 |
| ALL0053 | 2/M | *IL12RB1* | c.1897G>T/ p.Glu633Ter | 50.7 | rs772340282 | - | 0.00001967 | - | - | - | Immunodeficiency 30, AR | -* |
| ALL0060 | 40/M | *LPIN2* | c.480_483del/ p.Lys160AsnfsTer23 | 44.0 | - | - | - | - | - | - | Majeed syndrome, AR | -* |
| ALL0071 | 2/F | *CTC1* | c.2249dup/ p.Gly751ArgfsTer40 | 47.0 | - | - | - | - | - | - | Dyskeratosis congenital, AR | -* |
| ALL0021 | 2/M | *LIG4* | c.1271_1275del/ p.Lys424ArgfsTer20 | 42.6 | rs772226399 | 0.000156 | 0.000148 | 0.0003 | - | - | LIG4 syndrome, AR | 26762768 |

*Likely pathogenic by ACMG/AMP guideline; PVS1- Null variant (nonsense, frameshift, canonical +/-1 or 2 splice sites, initiation codon, single or multi-exon deletion) in a gene where loss of function (LOF) is a known mechanism of disease, PM - Absent from controls (or at extremely low frequency if recessive) in Exome Sequencing Project, 1000 Genomes or ExAC.

Abbreviations; AD, Autosomal dominant; AR, Autosomal recessive; dbSNP, The Single Nucleotide Polymorphism Database; ExAC, Exome Aggregation Consortium; F, Female; gnomAD, The Genom Agrregation Database; KRGDB, Korean Reference Genome Database; M, Male; PMID, PubMed identifier; SCID, Severe combined immunodeficiency.

| **Supplement Table S2. Somatic sequence variants identified in Korean ALL patients**. | | | | | | | |
| --- | --- | --- | --- | --- | --- | --- | --- |
| **Patients** | **Diagnosis** | **Gene** | **Accession** | **Nucleotide** | **%Variant** | **Pathway** | **COSMIC70** |
| ALL0002 | B-ALL | *PTPN11* | NM_002834.4 | c.179G>T | 11.3 | RAS pathway |  |
| ALL0002 | B-ALL | *SMC1A* | NM_006306.3 | c.1254+1G>A | 90.3 |  |  |
| ALL0002 | B-ALL | *FLT3* | NM_004119.2 | c.2503G>A | 15.3 | RAS pathway |  |
| ALL0002 | B-ALL | *CBL* | NM_005188.3 | c.1121T>C | 5.2 | RAS pathway |  |
| ALL0005 | B-ALL | *USH2A* | NM_206933.2 | c.10342G>A | 29.1 | Others |  |
| ALL0005 | B-ALL | *NRAS* | NM_002524.4 | c.35G>A | 6.5 | RAS pathway |  |
| ALL0007 | T-ALL | *DNMT3A* | NM_022552.4 | c.2525A>G | 41.0 | chromatin structure modifiers and epigenetic regulators |  |
| ALL0007 | T-ALL | *NRAS* | NM_002524.4 | c.38G>A | 24.9 | RAS pathway |  |
| ALL0007 | T-ALL | *NOTCH1* | NM_017617.3 | c.4721T>C | 31.5 | NOTCH pathway |  |
| ALL0007 | T-ALL | *DNMT3A* | NM_022552.4 | c.2525A>G | 79.1 | chromatin structure modifiers and epigenetic regulators |  |
| ALL0009 | B-ALL | *GNB1* | NM_002074.3 | c.346G>T | 17.1 | Others |  |
| ALL0009 | B-ALL | *GNB1* | NM_002074.3 | c.347G>T | 17.3 | Others |  |
| ALL0009 | B-ALL | *DNMT3A* | NM_022552.4 | c.2408+5G>A | 75.3 | chromatin structure modifiers and epigenetic regulators |  |
| ALL0010 | B-ALL | *DNMT3A* | NM_022552.4 | c.2711C>T | 43.6 | chromatin structure modifiers and epigenetic regulators |  |
| ALL0011 | B-ALL | *CBL* | NM_005188.3 | c.1096-1_1096insCGAAAA | 7.1 | RAS pathway |  |
| ALL0011 | B-ALL | *LRP1B* | NM_018557.2 | c.11127G>A | 49.8 | Others |  |
| ALL0030 | B-ALL | *NRAS* | NM_002524.4 | c.34G>A | 30.3 | RAS pathway |  |
| ALL0030 | B-ALL | *TP53* | NM_000546.5 | c.527G>T | 5.8 | Cell cycle and p53 signaling pathway |  |
| ALL0031 | T-ALL | *FBXW7* | NM_033632.3 | c.1394G>A | 36.1 | NOTCH pathway | ID=COSM117310,COSM117309,COSM22965,COSM1149856,COSM117308;OCCURENCE=24(large_intestine),1(biliary_tract),1(urinary_tract),2(small_intestine),9(endometrium),1(salivary_gland),2(ovary),34(haematopoietic_and_lymphoid_tissue),1(lung) |
| ALL0031 | T-ALL | *NOTCH1* | NM_017617.3 | c.4793G>C | 43.9 | NOTCH pathway | ID=COSM305943,COSM13053;OCCURENCE=25(haematopoietic_and_lymphoid_tissue) |
| ALL0031 | T-ALL | *ERCC4* | NM_005236.2 | c.2266G>A | 46.9 |  |  |
| ALL0031 | T-ALL | *BCL11B* | NM_138576.2 | c.1347_1349dupCAC | 39 | Transcriptional processes |  |
| ALL0031 | T-ALL | *PTPN11* | NM_002834.3 | c.1165A>C | 5.7 | RAS pathway |  |
| ALL0031 | T-ALL | *NBEAL2* | NM_015175.2 | c.6681G>C | 45 |  |  |
| ALL0034 | B-ALL | *FAT1* | NM_005245.3 | c.398C>G | 46.3 | Wnt signaling |  |
| ALL0034 | B-ALL | *UNC13B* | NM_006377.3 | c.1959-1G>T | 47.9 |  |  |
| ALL0035 | T-ALL | *IL7R* | NM_002185.3 | c.725_726insGGGGT | 7.5 | Jak-STAT signaling pathway |  |
| ALL0035 | T-ALL | *IL7R* | NM_002185.3 | c.725_726insGGGGTG | 5.2 | Jak-STAT signaling pathway |  |
| ALL0035 | T-ALL | *IL7R* | NM_002185.3 | c.728dupT | 7.3 | Jak-STAT signaling pathway |  |
| ALL0035 | T-ALL | *USP9X* | NM_001039590.2 | c.5141T>A | 69.0 | Others |  |
| ALL0035 | T-ALL | *PHF6* | NM_001015877.1 | c.560_561insCCCCCCC | 24.3 | chromatin structure modifiers and epigenetic regulators |  |
| ALL0035 | T-ALL | *EZH2* | NM_004456.4 | c.2233G>A | 33.6 | chromatin structure modifiers and epigenetic regulators | ID=COSM1087034,COSM1087033;OCCURENCE=2(endometrium) |
| ALL0035 | T-ALL | *RUNX1* | NM_001754.4 | c.494_495insGAGCCCCGG | 7.6 | Lymphoid development and differentiation |  |
| ALL0035 | T-ALL | *JAK1* | NM_002227.2 | c.2347C>T | 52.5 | Jak-STAT signaling pathway |  |
| ALL0035 | T-ALL | *CREBBP* | NM_004380.2 | c.6503G>A | 43.3 | chromatin structure modifiers and epigenetic regulators |  |
| ALL0036 | T-ALL | *BCL11B* | NM_138576.2 | c.2091delG | 28.1 | Transcriptional processes |  |
| ALL0036 | T-ALL | *FBXW7* | NM_033632.3 | c.1436G>A | 19.8 | NOTCH pathway | ID=COSM1154291,COSM447499,COSM22974,COSM447498,COSM94297;OCCURENCE=3(lung),15(haematopoietic_and_lymphoid_tissue),7(endometrium),1(breast),1(pancreas),1(kidney),13(large_intestine) |
| ALL0036 | T-ALL | *FBXW7* | NM_033632.3 | c.1394G>A | 54.1 | NOTCH pathway | ID=COSM117310,COSM117309,COSM22965,COSM1149856,COSM117308;OCCURENCE=24(large_intestine),1(biliary_tract),1(urinary_tract),2(small_intestine),9(endometrium),1(salivary_gland),2(ovary),34(haematopoietic_and_lymphoid_tissue),1(lung) |
| ALL0036 | T-ALL | *SBDS* | NM_016038.2 | c.127G>T | 6.9 |  |  |
| ALL0036 | T-ALL | *NOTCH1* | NM_017617.3 | c.4816_4818delTTC | 15.5 | NOTCH pathway |  |
| ALL0039 | B-ALL | *KRAS* | NM_004985.3 | c.38G>A | 14.8 | RAS pathway | ID=COSM1140132,COSM532;OCCURENCE=103(haematopoietic_and_lymphoid_tissue),2(pleura),5(cervix),17(gastrointestinal_tract_(site_indeterminate)),2(peritoneum),118(lung),23(thyroid),1(bone),4(upper_aerodigestive_tract),3(oesophagus),1(central_nervous_system),43(stomach),29(pancreas),3(liver),5(urinary_tract),1(genital_tract),3337(large_intestine),21(biliary_tract),23(prostate),17(breast),3(skin),19(small_intestine),50(endometrium),35(ovary),36(soft_tissue) |
| ALL0039 | B-ALL | *FLT3* | NM_004119.2 | c.1988A>G | 24.4 | RAS pathway | ID=COSM1366193;OCCURENCE=1(large_intestine) |
| ALL0040 | B-ALL | *KMT2D* | NM_003482.3 | c.15844C>T | 45.5 | chromatin structure modifiers and epigenetic regulators | ID=COSM221132,COSM221133;OCCURENCE=2(haematopoietic_and_lymphoid_tissue) |
| ALL0040 | B-ALL | *FLT3* | NM_004119.2 | c.2503G>A | 42.0 | RAS pathway | ID=COSM789;OCCURENCE=6(haematopoietic_and_lymphoid_tissue) |
| ALL0040 | B-ALL | *CREBBP* | NM_004380.2 | c.4463C>T | 46.9 | chromatin structure modifiers and epigenetic regulators | ID=COSM3356828;OCCURENCE=1(haematopoietic_and_lymphoid_tissue) |
| ALL0041 | B-ALL | *NRAS* | NM_002524.4 | c.35G>A | 43.1 | RAS pathway | ID=COSM564;OCCURENCE=60(skin),3(biliary_tract),49(large_intestine),1(genital_tract),1(kidney),1(pancreas),1(ovary),2(soft_tissue),4(endometrium),2(thyroid),1(lung),3(testis),324(haematopoietic_and_lymphoid_tissue),3(central_nervous_system),1(upper_aerodigestive_tract),2(NS) |
| ALL0046 | B-ALL | *NF1* | NM_001042492.2 | c.7549C>T | 5.9 | RAS pathway | ID=COSM133076;OCCURENCE=1(haematopoietic_and_lymphoid_tissue) |
| ALL0046 | B-ALL | *NF1* | NM_001042492.2 | c.2033dup | 5.7 | RAS pathway | ID=COSM1235317,COSM1382061;OCCURENCE=1(haematopoietic_and_lymphoid_tissue),2(large_intestine) |
| ALL0046 | B-ALL | *NRAS* | NM_002524.4 | c.35G>C | 20.3 | RAS pathway | ID=COSM565;OCCURENCE=1(lung),36(haematopoietic_and_lymphoid_tissue),1(testis),2(breast),12(skin),2(large_intestine) |
| ALL0047 | ETP | *GATA3* | NM_001002295.1 | c.431dup | 35.5 | Transcriptional processes |  |
| ALL0047 | ETP | *NOTCH1* | NM_017617.4 | c.7507C>T | 9.5 | NOTCH pathway | ID=COSM28669;OCCURENCE=7(haematopoietic_and_lymphoid_tissue) |
| ALL0047 | ETP | *SLC37A4* | NM_001164277.1 | c.1124+4A>G | 41.9 |  |  |
| ALL0047 | ETP | *IL7R* | NM_002185.3 | c.722_736del | 8.4 | Jak-STAT signaling pathway |  |
| ALL0047 | ETP | *GATA3* | NM_001002295.1 | c.869T>G | 38.7 | Transcriptional processes |  |
| ALL0047 | ETP | *ETV6* | NM_001987.4 | c.314G>T | 58.2 | Lymphoid development and differentiation |  |
| ALL0047 | ETP | *BRCA2* | NM_000059.3 | c.4714G>T | 39.0 |  |  |
| ALL0047 | ETP | *IL7R* | NM_002185.3 | c.736A>T | 7.3 | Jak-STAT signaling pathway |  |
| ALL0047 | ETP | *IKZF1* | NM_006060.5 | c.415C>T | 20.2 | Lymphoid development and differentiation |  |
| ALL0048 | B-ALL | *PTPN11* | NM_002834.4 | c.218C>T | 30.9 | RAS pathway | ID=COSM13019;OCCURENCE=12(haematopoietic_and_lymphoid_tissue) |
| ALL0048 | B-ALL | *CREBBP* | NM_004380.2 | c.2480_2481insCCGC | 39.1 | chromatin structure modifiers and epigenetic regulators |  |
| ALL0048 | B-ALL | *FLT3* | NM_004119.2 | c.2503G>T | 9.0 | RAS pathway | ID=COSM783;OCCURENCE=208(haematopoietic_and_lymphoid_tissue) |
| ALL0048 | B-ALL | *FLT3* | NM_004119.2 | c.2504_2506del | 5.5 | RAS pathway | ID=COSM27651;OCCURENCE=1(haematopoietic_and_lymphoid_tissue) |
| ALL0048 | B-ALL | *FLT3* | NM_004119.2 | c.1727T>C | 11.4 | RAS pathway |  |
| ALL0049 | B-ALL | *UNC13D* | NM_199242.2 | c.1299-4G>T | 48.0 | Transcriptional processes |  |
| ALL0050 | B-ALL | *NRAS* | NM_002524.4 | c.38G>A | 6.3 | RAS pathway | ID=COSM573;OCCURENCE=6(large_intestine),24(skin),1(endometrium),181(haematopoietic_and_lymphoid_tissue),1(thyroid),2(upper_aerodigestive_tract),1(NS) |
| ALL0050 | B-ALL | *KRAS* | NM_004985.4 | c.38G>A | 28.1 | RAS pathway | ID=COSM1140132,COSM532;OCCURENCE=103(haematopoietic_and_lymphoid_tissue),2(pleura),5(cervix),17(gastrointestinal_tract_(site_indeterminate)),2(peritoneum),118(lung),23(thyroid),1(bone),4(upper_aerodigestive_tract),3(oesophagus),1(central_nervous_system),43(stomach),29(pancreas),3(liver),5(urinary_tract),1(genital_tract),3337(large_intestine),21(biliary_tract),23(prostate),17(breast),3(skin),19(small_intestine),50(endometrium),35(ovary),36(soft_tissue) |
| ALL0050 | B-ALL | *FLT3* | NM_004119.2 | c.1715A>C | 14.5 | RAS pathway |  |
| ALL0054 | T-ALL | *CDKN1B* | NM_004064.3 | c.180G>A | 48.1 | Cell cycle and p53 signaling pathway |  |
| ALL0054 | T-ALL | *SETD2* | NM_014159.6 | c.7195_7196insAGACCTCTCCCATCAAATCCCAC | 9.8 | chromatin structure modifiers and epigenetic regulators |  |
| ALL0054 | T-ALL | *NOTCH1* | NM_017617.3 | c.7378G>T | 49.9 | NOTCH pathway | ID=COSM747569;OCCURENCE=1(haematopoietic_and_lymphoid_tissue) |
| ALL0054 | T-ALL | *ATM* | NM_000051.3 | c.2941C>T | 43.5 | Cell cycle and p53 signaling pathway |  |
| ALL0054 | T-ALL | *IL7R* | NM_002185.3 | c.756_757insCCTTGGGGT | 13.5 | Jak-STAT signaling pathway |  |
| ALL0054 | T-ALL | *STAT5B* | NM_012448.3 | c.2323C>T | 46.2 | Jak-STAT signaling pathway |  |
| ALL0054 | T-ALL | *NOTCH1* | NM_017617.3 | c.4754T>A | 38.4 | NOTCH pathway | ID=COSM308599;OCCURENCE=1(haematopoietic_and_lymphoid_tissue) |
| ALL0054 | T-ALL | *KMT2A* | NM_001197104.1 | c.370A>C | 46.9 | chromatin structure modifiers and epigenetic regulators |  |
| ALL0055 | B-ALL | *RAD21* | NM_006265.2 | c.319G>C | 18.5 | Others |  |
| ALL0056 | B-ALL | *FLT3* | NM_004119.2 | c.1794_1799dup | 14.9 | RAS pathway |  |
| ALL0058 | B-ALL | *GNB1* | NM_002074.4 | c.239T>A | 31.1 | Others |  |
| ALL0058 | B-ALL | *PAX5* | NM_016734.2 | c.547G>C | 51.6 | Lymphoid development and differentiation |  |
| ALL0063 | B-ALL | *ATG2B* | NM_018036.6 | c.2540+1G>A | 38.9 | Others |  |
| ALL0063 | B-ALL | *PFKM* | NM_001166686.1 | c.2475G>T | 39.1 | Others |  |
| ALL0065 | B-ALL | *SETD2* | NM_014159.6 | c.1413dup | 43.3 | chromatin structure modifiers and epigenetic regulators |  |
| ALL0065 | B-ALL | *IKZF1* | NM_006060.4 | c.1032dup | 27.6 | Lymphoid development and differentiation |  |
| ALL0065 | B-ALL | *CREBBP* | NM_004380.2 | c.4288T>G | 43.5 | chromatin structure modifiers and epigenetic regulators |  |
| ALL0065 | B-ALL | *NF1* | NM_001042492.2 | c.6215A>T | 13.4 | RAS pathway |  |
| ALL0065 | B-ALL | *SETD2* | NM_014159.6 | c.7540C>A | 19.6 | chromatin structure modifiers and epigenetic regulators |  |
| ALL0065 | B-ALL | *FLT3* | NM_004119.2 | c.1727T>A | 15.1 | RAS pathway | ID=COSM1197843;OCCURENCE=1(haematopoietic_and_lymphoid_tissue) |
| ALL0068 | B-ALL | *PAX5* | NM_016734.2 | c.621_633del | 5.4 | Lymphoid development and differentiation |  |
| ALL0068 | B-ALL | *PAX5* | NM_016734.2 | c.617del | 6.9 | Lymphoid development and differentiation |  |
| ALL0069 | B-ALL | *PTK2B* | NM_173174.2 | c.1798C>T | 29.7 | Others |  |
| ALL0070 | B-ALL | *CHD4* | NM_001273.3 | c.3484C>T | 44.0 | chromatin structure modifiers and epigenetic regulators | ID=COSM1476831,COSM942713,COSM942712;OCCURENCE=3(endometrium),1(large_intestine),1(breast) |
| ALL0072 | B-ALL | *CREBBP* | NM_004380.2 | c.4462C>A | 27.0 | chromatin structure modifiers and epigenetic regulators |  |
| ALL0072 | B-ALL | *CREBBP* | NM_004380.2 | c.3862C>T | 31.5 | chromatin structure modifiers and epigenetic regulators |  |
| ALL0072 | B-ALL | *SETD2* | NM_014159.6 | c.548T>G | 6.5 | chromatin structure modifiers and epigenetic regulators |  |
| ALL0073 | B-ALL | *ETV6* | NM_001987.4 | c.1297C>T | 17.1 | Lymphoid development and differentiation |  |
| ALL0073 | B-ALL | *FLT3* | NM_004119.2 | c.1780T>G | 16.1 | RAS pathway |  |
| ALL0090 | B-ALL | *NRAS* | NM_002524.4 | c.34G>A | 5.9 | RAS pathway | ID=COSM563;OCCURENCE=6(large_intestine),6(prostate),13(skin),1(soft_tissue),93(haematopoietic_and_lymphoid_tissue),3(lung),21(upper_aerodigestive_tract) |
| ALL0093 | B-ALL | *CNOT3* | NM_014516.3 | c.1195G>T | 35.1 | Translation and RNA stability |  |
| ALL0093 | B-ALL | *ASXL1* | NM_015338.5 | c.1934del | 35.1 | chromatin structure modifiers and epigenetic regulators | ID=COSM6191274;OCCURENCE=1(Haematopoietic and lymphoid tissue) |
| ALL0093 | B-ALL | *SETBP1* | NM_015559.2 | c.2632A>C | 33.5 | chromatin structure modifiers and epigenetic regulators |  |
| ALL0006 | B-ALL | *ASXL1* | NM_015338.5 | c.1898dup | 48.2 | chromatin structure modifiers and epigenetic regulators |  |
| ALL0043 | ETP | *WT1* | NM_024426.4 | c.822_823insTCCCT | 32.5 | Transcriptional processes |  |
| ALL0044 | B-ALL | *NRAS* | NM_002524.4 | c.38G>A | 42.9 | RAS pathway | ID=COSM573;OCCURENCE=6(large_intestine),24(skin),1(endometrium),181(haematopoietic_and_lymphoid_tissue),1(thyroid),2(upper_aerodigestive_tract),1(NS) |
| ALL0044 | B-ALL | *ABL2* | NM_007314.3 | c.2177A>T | 5.5 | ABL |  |
| ALL0003 | B-ALL | *LAPTM5* | NM_006762.2 | c.259-8_259-2del | 15.1 | Others |  |
| ALL0003 | B-ALL | *LAPTM5* | NM_006762.2 | c.259-10T>G | 17.3 | Others |  |
| ALL0008 | B-ALL | *SH2B3* | NM_001291424.1 | c.659dup | 34.1 | Jak-STAT signaling pathway |  |
| ALL0008 | B-ALL | *NRAS* | NM_002524.4 | c.34G>A | 7.4 | RAS pathway | ID=COSM563;OCCURENCE=6(large_intestine),6(prostate),13(skin),1(soft_tissue),93(haematopoietic_and_lymphoid_tissue),3(lung),21(upper_aerodigestive_tract) |
| ALL0081 | T-ALL | *WT1* | NM_024426.4 | c.1288C>T | 81.6 | Transcriptional processes | ID=COSM21401;OCCURENCE=2(kidney) |
| ALL0081 | T-ALL | *CBL* | NM_005188.3 | c.1217_1227del | 48.4 | RAS pathway |  |
| ALL0081 | T-ALL | *NOTCH1* | NM_017617.4 | c.7326_7327insCTTTGAC | 12.5 | NOTCH pathway |  |
| ALL0081 | T-ALL | *NOTCH1* | NM_017617.4 | c.4775T>C | 41.2 | NOTCH pathway | ID=COSM13050;OCCURENCE=7(haematopoietic_and_lymphoid_tissue) |
| ALL0081 | T-ALL | *NOTCH1* | NM_017617.4 | c.4774T>C | 41.2 | NOTCH pathway | ID=COSM308613,COSM1461173;OCCURENCE=1(large_intestine) |
| ALL0081 | T-ALL | *TPI1* | NM_001159287.1 | c.792_794dup | 37.3 | Others |  |
| ALL0081 | T-ALL | *JAK1* | NM_002227.2 | c.2108G>T | 37.4 | Jak-STAT signaling pathway | ID=COSM305942;OCCURENCE=2(haematopoietic_and_lymphoid_tissue),3(liver) |
| ALL0081 | T-ALL | *GATA3* | NM_001002295.1 | c.827G>A | 41.0 | Transcriptional processes | ID=COSM294402;OCCURENCE=2(haematopoietic_and_lymphoid_tissue),1(large_intestine) |
| ALL0081 | T-ALL | *CBL* | NM_005188.3 | c.1217C>G | 13.0 | RAS pathway |  |
| ALL0081 | T-ALL | *CBL* | NM_005188.3 | c.1227+3_1227+9del | 47.6 | RAS pathway |  |
| ALL0081 | T-ALL | *CHD4* | NM_001273.2 | c.3440A>T | 22.1 | chromatin structure modifiers and epigenetic regulators |  |
| ALL0081 | T-ALL | *TET3* | NM_001287491.1 | c.5063G>A | 38.3 | chromatin structure modifiers and epigenetic regulators |  |
| ALL0089 | B-ALL | *IDH2* | NM_001289910.1 | c.263G>A | 45.1 | chromatin structure modifiers and epigenetic regulators | ID=COSM41590;OCCURENCE=417(haematopoietic_and_lymphoid_tissue),1(NS) |
| ALL0089 | B-ALL | *GATA3* | NM_001002295.1 | c.857A>G | 63.6 | Transcriptional processes |  |
| ALL0089 | B-ALL | *PAX5* | NM_016734.2 | c.77T>G | 36.1 | Lymphoid development and differentiation | ID=COSM85951;OCCURENCE=2(large_intestine),1(liver),3(haematopoietic_and_lymphoid_tissue),1(oesophagus) |
| ALL0037 | B-ALL | *CREBBP* | NM_004380.2 | c.5499C>A | 6.5 | chromatin structure modifiers and epigenetic regulators |  |
| ALL0037 | B-ALL | *NRAS* | NM_002524.4 | c.35G>C | 45.1 | RAS pathway | ID=COSM565;OCCURENCE=1(lung),36(haematopoietic_and_lymphoid_tissue),1(testis),2(breast),12(skin),2(large_intestine) |
| ALL0037 | B-ALL | *AKT2* | NM_001626.5 | c.235C>A | 7.7 | PI3K-AKT-mTOR signaling pathway |  |
| ALL0038 | B-ALL | *MSH4* | NM_002440.3 | c.1285G>T | 39.5 | Others |  |
| ALL0038 | B-ALL | *XBP1* | NM_005080.3 | c.599+1_599+2dup | 44.9 | Lymphoid development and differentiation |  |
| ALL0038 | B-ALL | *SRP72* | NM_006947.3 | c.550T>C | 26.2 | Others |  |
| ALL0075 | B-ALL | *PTPN11* | NM_002834.3 | c.181G>T | 50.9 | RAS pathway | ID=COSM13011;OCCURENCE=33(haematopoietic_and_lymphoid_tissue),1(autonomic_ganglia),1(large_intestine) |
| ALL0075 | B-ALL | *FAT1* | NM_005245.3 | c.1333G>T | 36.9 | Wnt signaling |  |
| ALL0075 | B-ALL | *CUX1* | NM_001913.3 | c.1769G>T | 7.1 | Others |  |
| ALL0078 | T-ALL | *NF1* | NM_001042492.2 | c.2834_2835dup | 26.6 | RAS pathway |  |
| ALL0078 | T-ALL | *KMT2C* | NM_170606.2 | c.11101A>G | 27.4 | chromatin structure modifiers and epigenetic regulators |  |
| ALL0078 | T-ALL | *DGKH* | NM_178009.4 | c.623-8_623-3dup | 100.0 | Others |  |
| ALL0082 | T-ALL | *NRAS* | NM_002524.4 | c.182A>G | 24.8 | RAS pathway | ID=COSM584;OCCURENCE=4(soft_tissue),3(ovary),4(endometrium),474(skin),7(breast),1(small_intestine),7(urinary_tract),7(adrenal_gland),1(liver),4(eye),2(pancreas),4(biliary_tract),49(large_intestine),5(central_nervous_system),49(NS),289(thyroid),12(lung),9(meninges),3(testis),69(haematopoietic_and_lymphoid_tissue) |
| ALL0082 | T-ALL | *DNMT3A* | NM_022552.4 | c.2644C>T | 23.2 | chromatin structure modifiers and epigenetic regulators | ID=COSM1166704,COSM53042;OCCURENCE=161(haematopoietic_and_lymphoid_tissue) |
| ALL0082 | T-ALL | *PTEN* | NM_000314.6 | c.739_740insGCCCG | 8.3 | PI3K-AKT-mTOR signaling pathway |  |
| ALL0084 | B-ALL | *EBF1* | NM_001324101.1 | c.1048G>A | 23.8 | Lymphoid development and differentiation |  |
| ALL0085 | T-ALL | *NRAS* | NM_002524.4 | c.182A>G | 46.4 | RAS pathway | ID=COSM584;OCCURENCE=4(soft_tissue),3(ovary),4(endometrium),474(skin),7(breast),1(small_intestine),7(urinary_tract),7(adrenal_gland),1(liver),4(eye),2(pancreas),4(biliary_tract),49(large_intestine),5(central_nervous_system),49(NS),289(thyroid),12(lung),9(meninges),3(testis),69(haematopoietic_and_lymphoid_tissue) |
| ALL0085 | T-ALL | *PTEN* | NM_000314.6 | c.739_740insGCCCG | 16.8 | PI3K-AKT-mTOR signaling pathway |  |
| ALL0085 | T-ALL | *FBXW7* | NM_033632.3 | c.584+1G>A | 97.8 | NOTCH pathway |  |
| ALL0085 | T-ALL | *PHF6* | NM_001015877.1 | c.420del | 11.3 | chromatin structure modifiers and epigenetic regulators |  |
| ALL0085 | T-ALL | *PHF6* | NM_001015877.1 | c.422_437del | 10.2 | chromatin structure modifiers and epigenetic regulators |  |
| ALL0085 | T-ALL | *CREBBP* | NM_004380.2 | c.4262G>T | 23.5 | chromatin structure modifiers and epigenetic regulators |  |
| ALL0085 | T-ALL | *PHF6* | NM_001015877.1 | c.437T>G | 8.1 | chromatin structure modifiers and epigenetic regulators |  |
| ALL0085 | T-ALL | *ANK1* | NM_001142446.1 | c.755T>A | 22.6 | Others |  |
| ALL0087 | B-ALL | *TP53* | NM_000546.5 | c.743G>T | 8.0 | Cell cycle and p53 signaling pathway | ID=COSM241995,COSM1646857,COSM241994,COSM6549,COSM241996,COSM3378346;OCCURENCE=1(stomach),1(central_nervous_system),1(thymus),11(upper_aerodigestive_tract),3(oesophagus),30(lung),3(haematopoietic_and_lymphoid_tissue),2(ovary),6(breast),2(skin),1(kidney),1(pancreas),3(liver),3(urinary_tract),4(large_intestine),1(prostate) |
| ALL0087 | B-ALL | *PAX5* | NM_016734.2 | c.963dup | 14.6 | Lymphoid development and differentiation | ID=COSM85890;OCCURENCE=3(haematopoietic_and_lymphoid_tissue) |
| ALL0088 | B-ALL | *PTPN11* | NM_002834.3 | c.417G>T | 21.3 | RAS pathway |  |
| ALL0088 | B-ALL | *KMT2D* | NM_003482.3 | c.16372G>T | 42.1 | chromatin structure modifiers and epigenetic regulators |  |
| ALL0088 | B-ALL | *SETD2* | NM_014159.6 | c.6036_6037insGG | 43.1 | chromatin structure modifiers and epigenetic regulators |  |
| ALL0088 | B-ALL | *SETD2* | NM_014159.6 | c.5159A>C | 10.5 | chromatin structure modifiers and epigenetic regulators |  |
| ALL0088 | B-ALL | *ZFHX4* | NM_024721.4 | c.2353C>T | 20.0 | DNA repair |  |
| ALL0080 | B-ALL | *NRAS* | NM_002524.4 | c.38G>A | 7.2 | RAS pathway | ID=COSM573;OCCURENCE=6(large_intestine),24(skin),1(endometrium),181(haematopoietic_and_lymphoid_tissue),1(thyroid),2(upper_aerodigestive_tract),1(NS) |
| ALL0080 | B-ALL | *NRAS* | NM_002524.4 | c.35G>A | 25.9 | RAS pathway | ID=COSM564;OCCURENCE=60(skin),3(biliary_tract),49(large_intestine),1(genital_tract),1(kidney),1(pancreas),1(ovary),2(soft_tissue),4(endometrium),2(thyroid),1(lung),3(testis),324(haematopoietic_and_lymphoid_tissue),3(central_nervous_system),1(upper_aerodigestive_tract),2(NS) |
| ALL0080 | B-ALL | *PAX5* | NM_016734.2 | c.77T>G | 11.6 | Lymphoid development and differentiation | ID=COSM85951;OCCURENCE=2(large_intestine),1(liver),3(haematopoietic_and_lymphoid_tissue),1(oesophagus) |
| ALL0028 | B-ALL | *EZH2* | NM_004456.4 | c.1538_1539dupTG | 37.7 | chromatin structure modifiers and epigenetic regulators |  |
| ALL0029 | B-ALL | *ETV6* | NM_001987.4 | c.140_141insCGGGGGTC | 6.3 | Lymphoid development and differentiation |  |
| ALL0029 | B-ALL | *SETD2* | NM_014159.6 | c.5733_5734insAAGGGGGA | 5.8 | chromatin structure modifiers and epigenetic regulators |  |
| ALL0029 | B-ALL | *SETD2* | NM_014159.6 | c.5730T>A | 7.6 | chromatin structure modifiers and epigenetic regulators |  |
| ALL0029 | B-ALL | *TOX* | NM_014729.2 | c.-7_-6insCG | 20.8 | Others |  |
| ALL0029 | B-ALL | *TOX* | NM_014729.2 | c.-8A>G | 20.8 | Others |  |
| ALL0032 | B-ALL | *NRAS* | NM_002524.4 | c.35G>C | 7.5 | RAS pathway | ID=COSM565;OCCURENCE=1(lung),36(haematopoietic_and_lymphoid_tissue),1(testis),2(breast),12(skin),2(large_intestine) |
| ALL0032 | B-ALL | *FLT3* | NM_004119.2 | c.2028C>A | 20.0 | RAS pathway | ID=COSM303886;OCCURENCE=2(haematopoietic_and_lymphoid_tissue) |
| ALL0032 | B-ALL | *VWF* | NM_000552.3 | c.4790G>A | 8.1 | Others |  |
| ALL0001 | B-ALL | *KRAS* | NM_004985.3 | c.38G>A | 34.4 | RAS pathway | ID=COSM1140132,COSM532;OCCURENCE=103(haematopoietic_and_lymphoid_tissue),2(pleura),5(cervix),17(gastrointestinal_tract_(site_indeterminate)),2(peritoneum),118(lung),23(thyroid),1(bone),4(upper_aerodigestive_tract),3(oesophagus),1(central_nervous_system),43(stomach),29(pancreas),3(liver),5(urinary_tract),1(genital_tract),3337(large_intestine),21(biliary_tract),23(prostate),17(breast),3(skin),19(small_intestine),50(endometrium),35(ovary),36(soft_tissue) |
| ALL0001 | B-ALL | *VWF* | NM_000552.3 | c.8332C>T | 40.4 | Others | ID=COSM376165;OCCURENCE=1(lung),1(oesophagus) |
| ALL0004 | B-ALL | *BRCC3* | NM_024332.3 | c.410G>A | 37.9 | Others | ID=COSM457148;OCCURENCE=1(breast) |
| ALL0012 | B-ALL | *FLT3* | NM_004119.2 | c.1753T>C | 5.1 | RAS pathway |  |
| ALL0013 | B-ALL | *VWF* | NM_000552.3 | c.80G>A | 21.2 | Others |  |
| ALL0016 | B-ALL | *ETV6* | NM_001987.4 | c.1169_1170insTT | 6.6 | Lymphoid development and differentiation |  |
| ALL0016 | B-ALL | *ASXL1* | NM_015338.5 | c.1934dup | 5.5 | chromatin structure modifiers and epigenetic regulators | ID=COSM1411076;OCCURENCE=1(large_intestine) |
| ALL0017 | B-ALL | *NRAS* | NM_002524.4 | c.181C>A | 15.6 | RAS pathway | ID=COSM580;OCCURENCE=1(prostate),79(large_intestine),1(biliary_tract),2(urinary_tract),2(eye),1(kidney),2(liver),2(pancreas),2(breast),437(skin),2(endometrium),1(ovary),15(soft_tissue),5(autonomic_ganglia),2(cervix),71(haematopoietic_and_lymphoid_tissue),1(meninges),56(thyroid),18(lung),29(NS),4(bone),4(central_nervous_system),1(stomach) |
| ALL0017 | B-ALL | *KRAS* | NM_004985.4 | c.437C>T | 7.5 | RAS pathway | ID=COSM19900,COSM1360827;OCCURENCE=2(thyroid),1(haematopoietic_and_lymphoid_tissue),18(large_intestine) |
| ALL0017 | B-ALL | *KRAS* | NM_004985.4 | c.436G>A | 12.8 | RAS pathway | ID=COSM1165198,COSM19404;OCCURENCE=91(large_intestine),2(small_intestine),1(upper_aerodigestive_tract),11(haematopoietic_and_lymphoid_tissue) |
| ALL0017 | B-ALL | *SF3A1* | NM_005877.5 | c.1091_1093del | 7.8 | Others |  |
| ALL0018 | B-ALL | *SETD2* | NM_014159.6 | c.7483A>T | 11.1 | chromatin structure modifiers and epigenetic regulators |  |
| ALL0018 | B-ALL | *GNB1* | NM_002074.3 | c.265A>C | 6.5 | Others |  |
| ALL0018 | B-ALL | *FLT3* | NM_004119.2 | c.1419-4dup | 5.6 | RAS pathway |  |
| ALL0021 | B-ALL | *NRAS* | NM_002524.4 | c.38G>T | 12.3 | RAS pathway | ID=COSM574;OCCURENCE=1(soft_tissue),2(large_intestine),17(skin),1(central_nervous_system),41(haematopoietic_and_lymphoid_tissue) |
| ALL0021 | B-ALL | *GNB1* | NM_002074.3 | c.300_301insGGGTCCTCCTGGGTC | 11.9 | Others |  |
| ALL0021 | B-ALL | *LRP1B* | NM_018557.2 | c.5266A>T | 10.7 | Others |  |
| ALL0022 | B-ALL | *SETD2* | NM_014159.6 | c.1366C>T | 23.1 | chromatin structure modifiers and epigenetic regulators |  |
| ALL0022 | B-ALL | *SETD2* | NM_014159.6 | c.208C>T | 22.5 | chromatin structure modifiers and epigenetic regulators |  |
| ALL0022 | B-ALL | *IKZF3* | NM_012481.4 | c.536G>A | 6.5 | Lymphoid development and differentiation |  |
| ALL0023 | B-ALL | *TP53* | NM_000546.5 | c.527G>T | 8.4 | Cell cycle and p53 signaling pathway | ID=COSM117395,COSM117398,COSM117396,COSM3378352,COSM1640850,COSM117397,COSM10645;OCCURENCE=2(prostate),19(large_intestine),2(genital_tract),1(adrenal_gland),7(urinary_tract),5(liver),4(pancreas),8(breast),1(skin),6(ovary),1(haematopoietic_and_lymphoid_tissue),15(lung),20(oesophagus),21(upper_aerodigestive_tract),3(bone),3(central_nervous_system),10(stomach) |
| ALL0024 | B-ALL | *PIK3R1* | NM_181523.2 | c.1430_1431insAAAACC | 20.1 | PI3K-AKT-mTOR signaling pathway |  |
| ALL0025 | B-ALL | *GNB1* | NM_002074.4 | c.239T>G | 34.0 | Others |  |
| ALL0025 | B-ALL | *USH2A* | NM_206933.2 | c.5381C>T | 22.6 | Others |  |
| ALL0025 | B-ALL | *IL3RA* | NM_002183.3 | c.166G>A | 56.1 | Jak-STAT signaling pathway |  |
| ALL0025 | B-ALL | *HUWE1* | NM_031407.5 | c.7948C>T | 24.0 | Others |  |
| ALL0061 | B-ALL | *PBX1* | NM_002585.3 | c.395C>A | 45.6 | Others |  |
| ALL0057 | B-ALL | *KRAS* | NM_004985.4 | c.35G>A | 28.1 | RAS pathway | ID=COSM521,COSM1135366;OCCURENCE=1(salivary_gland),171(endometrium),265(ovary),20(soft_tissue),19(urinary_tract),1(kidney),2323(pancreas),15(liver),3(eye),22(prostate),321(biliary_tract),6237(large_intestine),18(breast),24(skin),58(small_intestine),25(upper_aerodigestive_tract),10(oesophagus),4(central_nervous_system),93(stomach),2(testis),19(cervix),114(haematopoietic_and_lymphoid_tissue),15(gastrointestinal_tract_(site_indeterminate)),37(thyroid),737(lung),25(peritoneum) |
| ALL0091 | B-ALL | *KRAS* | NM_004985.3 | c.68T>G | 43.5 | RAS pathway | ID=COSM303853;OCCURENCE=1(haematopoietic_and_lymphoid_tissue) |
| ALL0060 | B-ALL | *KRAS* | NM_004985.4 | c.34G>A | 5.9 | RAS pathway |  |
| ALL0060 | B-ALL | *NRAS* | NM_002524.4 | c.34G>A | 8.4 | RAS pathway |  |

| **Supplement Table S3. Genes included in the hematologic cancer panel.** |
| --- |
| *ABCB1, ABCB7, ABCG2, ABCG5, ABCG8, ABL1, ABL2, ACD, ACTB, ACTN1, ADA, ADAMTS13, AIRE, AK1, AK2, AKT2, ALAS2, ALDOA, AMN, ANK1, ANKRD26, AP3B1, ARID1A, ARPC1B, ASXL1, ATG2B, ATM, ATR, ATRX, AXIN1, BCL11B, BCL2, BCL6, BCOR, BCORL1, BHLHE41, BIRC3, BLM, BPGM, BRAF, BRCA1, BRCA2, BRCC3, BRINP3, BRIP1, BTG1, BTK, BTLA, C3, C4BPA, C4BPB, CALN1, CALR, CARD11, CASP10, CBL, CBLB, CBLC, CCND1, CD200, CD247, CD27, CD36, CD3D, CD3E, CD40LG, CD46, CD58, CD59, CD79B, CDAN1, CDKN1B, CDKN2A, CDKN2B, CEBPA, CFB, CFH, CFHR1, CFHR3, CFHR4, CFHR5, CFI, CHD1, CHD4, CHD9, CHMP2B, CLPB, CNOT3, COX4I2, CREBBP, CRLF2, CSF1R, CSF2RA, CSF3R, CTC1, CTCF, CTSC, CUBN, CUX1, CXCR4, CYB5R3, CYBA, CYBB, CYCS, DCLRE1C, DDX41, DGKE, DGKH, DHFR, DIS3, DKC1, DNM2, DNMT1, DNMT3A, EBF1, ECT2L, EED, EGFR, EGLN1, EGLN2, EGLN3, EHMT1, ELANE, EP300, EPAS1, EPB41, EPB42, EPCAM, EPO, EPOR, ERCC4, ERG, ETNK1, ETV6, EZH2, F2R, FANCA, FANCB, FANCC, FANCD2, FANCE, FANCF, FANCG, FANCI, FANCL, FANCM, FAS, FASLG, FAT1, FBXW7, FCGR1A, FCGR3B, FERMT3, FLI1, FLNA, FLT3, FOXP3, G6PC3, G6PD, GATA1, GATA2, GATA3, GCLC, GFI1, GFI1B, GIF, GINS1, GLRX5, GNAS, GNB1, GP1BA, GP1BB, GP9, GPI, GPRC5A, GPX1, GSKIP, GSN, GSR, GSS, HAX1, HBA1, HBA2, HBB, HBD, HCLS1, HFE, HIF1A, HIF1AN, HIF3A, HK1, HNRNPK, HOOK1, HOXA10, HOXA11, HRAS, HSPA9, HUWE1, ID3, IDH1, IDH2, IFNG, IFNGR1, IFNGR2, IKZF1, IKZF2, IKZF3, IL12RB1, IL2RB, IL2RG, IL3RA, IL7R, IRF1, ITGA2, ITGA2B, ITGB2, ITGB3, ITK, ITPKB, JAGN1, JAK1, JAK2, JAK3, JAKMIP2, JMJD1C, KDM5C, KDM6A, KDM7A, KIF23, KIT, KLF1, KMT2A, KMT2C, KMT2D, KRAS, LAMB4, LAMTOR2, LAPTM5, LCK, LEF1, LIG4, LMNA, LMO1, LMO2, LPIN2, LRP1B, LRRC4, LUC7L2, LYL1, LYST, MAD2L2, MAGT1, MAP2K1, MAP2K2, MASTL, MBL2, MECOM, MED13, MEF2B, MEF2C, MEFV, MET, MLH1, MLLT10, MLLT3, MPL, MRE11, MSH2, MSH4, MSH6, MTA1, MTAP, MTR, MTRR, MVK, MYB, MYC, MYD88, MYH9, MYSM1, NAF1, NBEAL2, NBN, NCF2, NCOR2, NF1, NHEJ1, NHP2, NLRP3, NOD2, NOP10, NOTCH1, NOTCH2, NPM1, NR3C1, NRAS, NT5C2, NT5C3A, NTRK3, NUP214, OS9, P2RY2, PALB2, PARN, PAX5, PBX1, PC, PCDHB1, PDGFRA, PDGFRB, PDHA1, PDHX, PFKL, PFKM, PGK1, PGM3, PHF6, PICALM, PIEZO1, PIGA, PIK3CD, PIK3R1, PKLR, PML, PMS2, PNP, POT1, PRDM1, PRF1, PRKACG, PRPF40B, PTCH2, PTEN, PTK2B, PTPN11, PTPN2, PTPRC, PTPRD, PUS1, RAB27A, RAC1, RAC2, RAD21, RAD50, RAD51, RAD51C, RAF1, RAG1, RAG2, RB1, RBBP6, RBM8A, RELN, RFWD3, RHAG, RHOA, RIT1, RMRP, RNF168, RPL10, RPL11, RPL15, RPL23, RPL26, RPL27, RPL31, RPL35A, RPL36, RPL5, RPS10, RPS14, RPS15, RPS17, RPS19, RPS24, RPS26, RPS27, RPS27A, RPS28, RPS29, RPS7, RTEL1, RUNX1, RUNX1T1, SAMD9L, SBDS, SBF2, SEC23B, SERPING1, SETBP1, SETD2, SF1, SF3A1, SF3B1, SH2B3, SH2D1A, SHOC2, SLC11A2, SLC19A2, SLC25A38, SLC2A1, SLC35C1, SLC37A4, SLC4A1, SLCO1B1, SLCO1B3, SLFN14, SLX4, SMARCD2, SMC1A, SMC3, SOS1, SPINK5, SPRED1, SPTA1, SPTB, SRC, SRCAP, SRP72, SRSF2, STAG1, STAG2, STAT3, STAT5B, STEAP3, STX11, STXBP2, SUZ12, SYNE1, TAL1, TAL2, TAZ, TBL1XR1, TBX1, TCF3, TCIRG1, TEC, TERC, TERF1, TERF2, TERF2IP, TERT, TET1, TET2, TET3, THBD, THPO, TINF2, TLX1, TLX3, TMPRSS6, TNFAIP3, TNFRSF13B, TNFRSF14, TNFRSF1A, TOX, TP53, TPI1, TPMT, TRAF3, TRNT1, TSLP, TSR2, TUBB1, TYK2, U2AF1, U2AF2, UBE2T, UGT1A1, UGT1A7, UNC13B, UNC13D, UNC5D, USB1, USH2A, USP9X, VHL, VPS13B, VPS45, VWF, WAS, WDR1, WIPF1, WRAP53, WT1, XBP1, XIAP, XK, XRCC2, YARS2, ZAP70, ZFHX4, ZNF197, ZRSR2* |


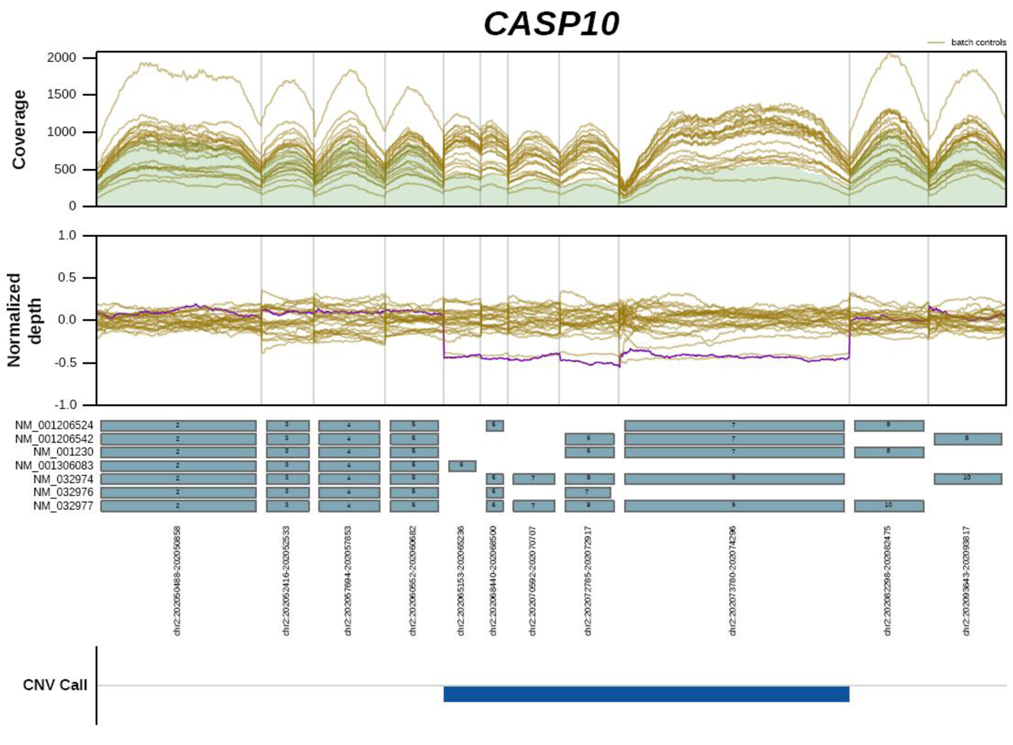


Supplement Fig. S1. *CASP10* gene deletion detected by the next generation sequencing (NGS) copy number variation (CNV) analysis in a patient with B-cell acute lymphoblastic leukemia/lymphoma (B-ALL).


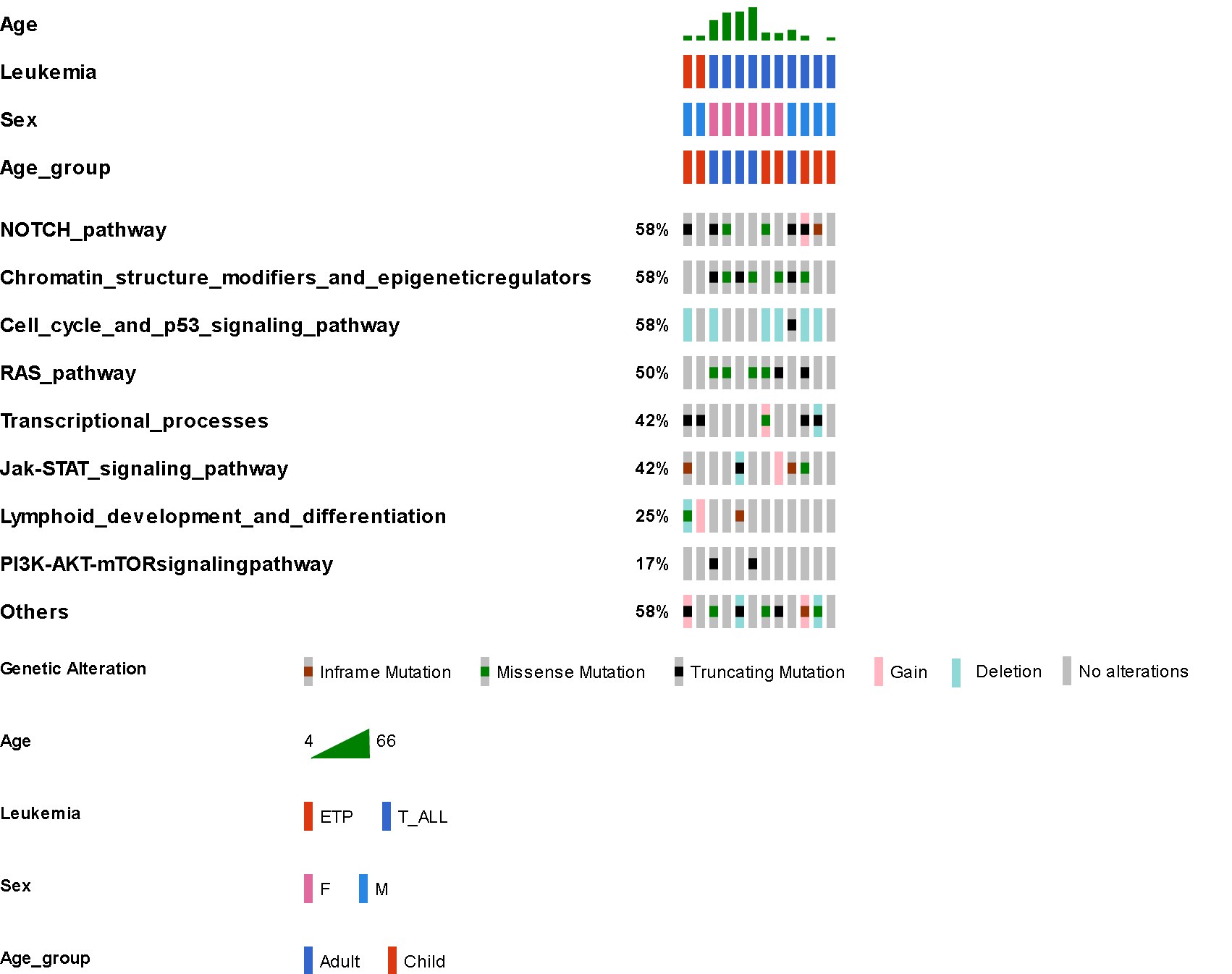


Supplement Fig. S2. Somatic gene alteration according to gene pathway in T-cell acute lymphoblastic leukemia/lymphoma (T-ALL). Data were analyzed by OncoPrinter (cBioPortal Version 1.14.0, Gao et al.Sci. Signal. 2013 and Cerami et al. Cancer Discov. 2012).


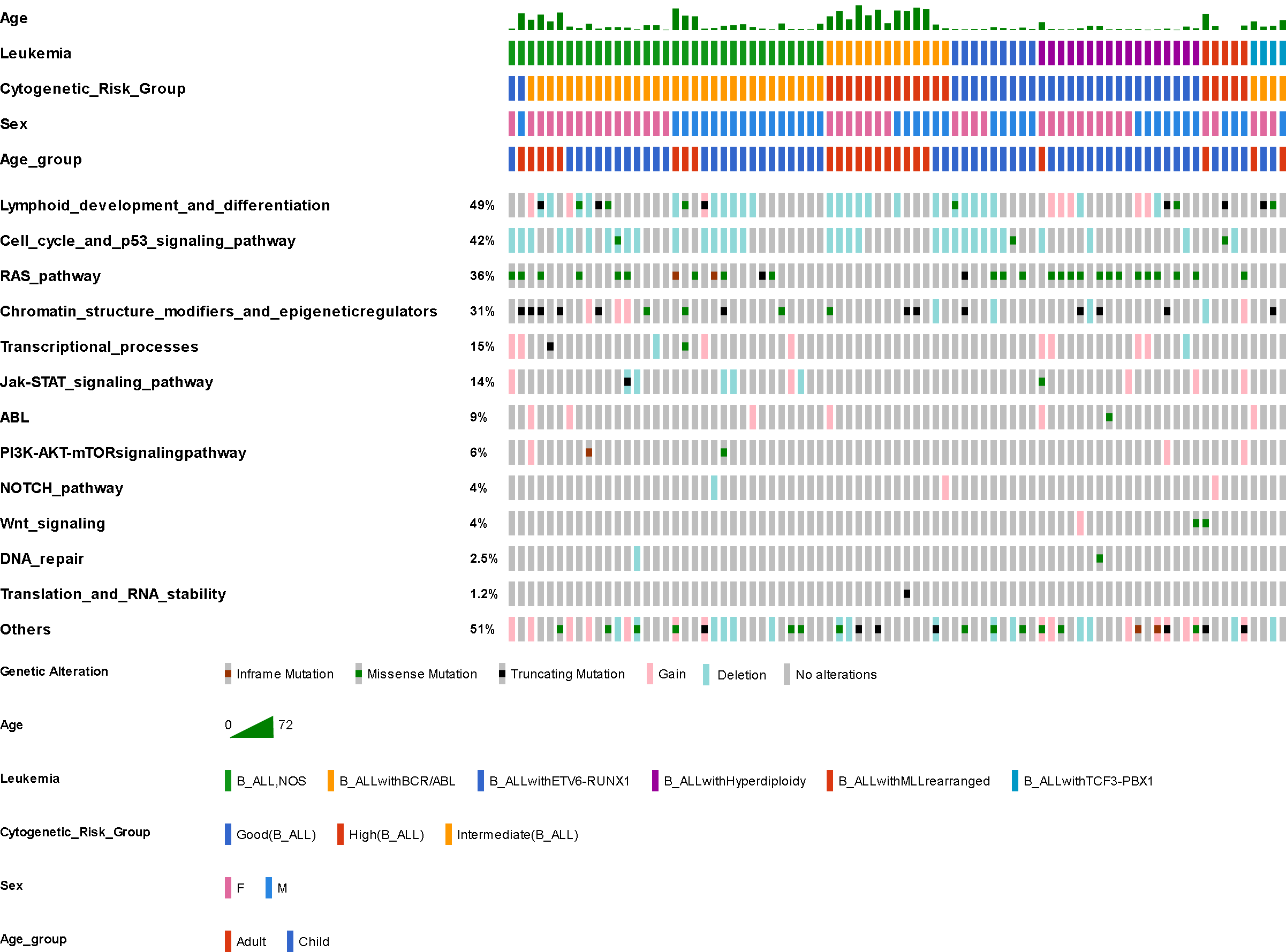


Supplement Fig. S3. Somatic gene alteration according to gene pathway in B-cell acute lymphoblastic leukemia/lymphoma (B-ALL). Data were analyzed by OncoPrinter (cBioPortal Version 1.14.0, Gao et al.Sci. Signal. 2013 and Cerami et al. Cancer Discov. 2012).


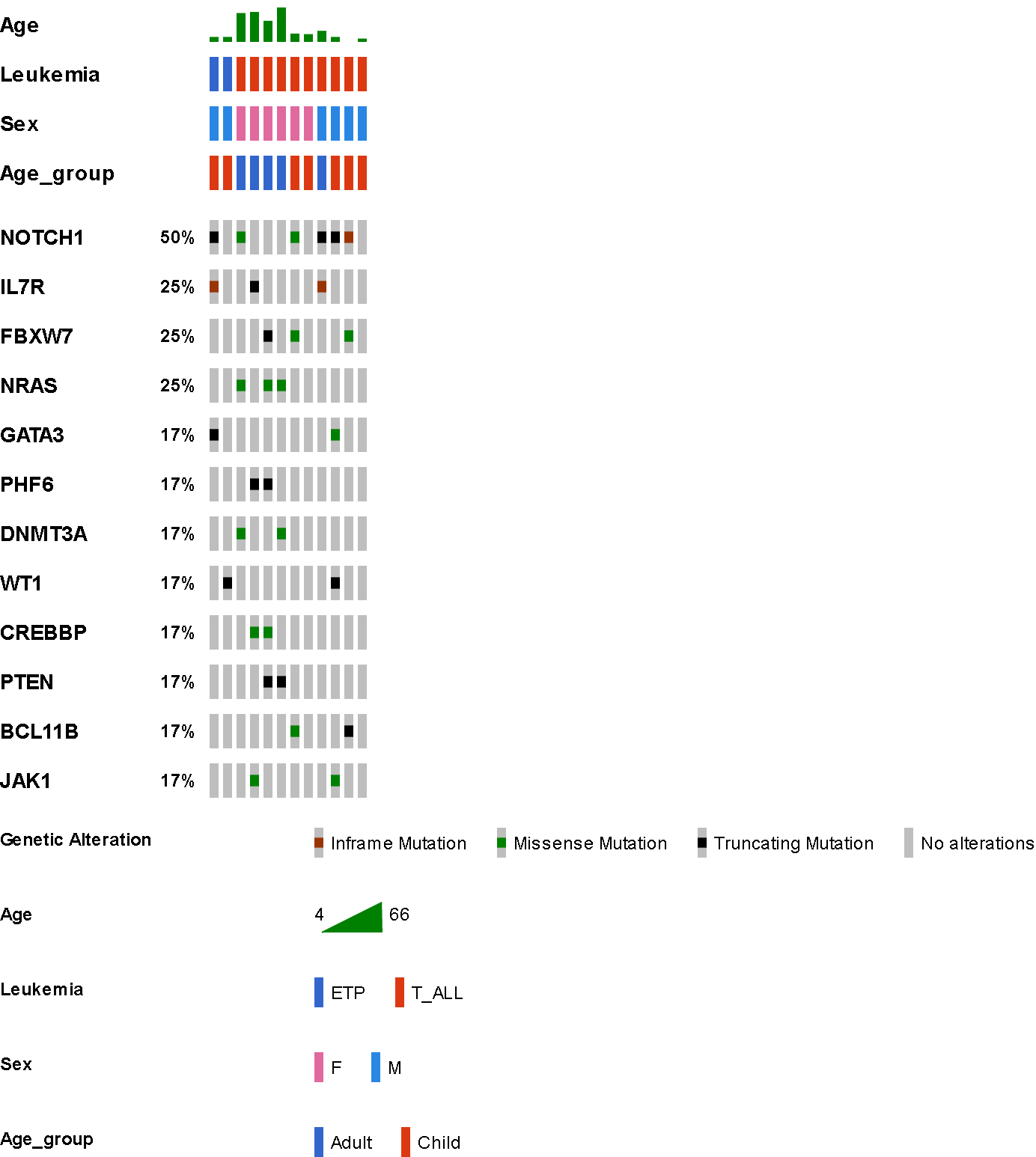


Supplement Fig. S4. Common sequence variants in T-cell acute lymphoblastic leukemia/lymphoma (T-ALL). Only ≥2 genes were shown in figure. **Truncating mutations** (nonsense, frameshift deletion, frameshift insertion, splice site); ****i**nframe** (inframe deletion, inframe insertion). Data were analyzed by OncoPrinter (cBioPortal Version 1.14.0, Gao et al.Sci. Signal. 2013 and Cerami et al. Cancer Discov. 2012).


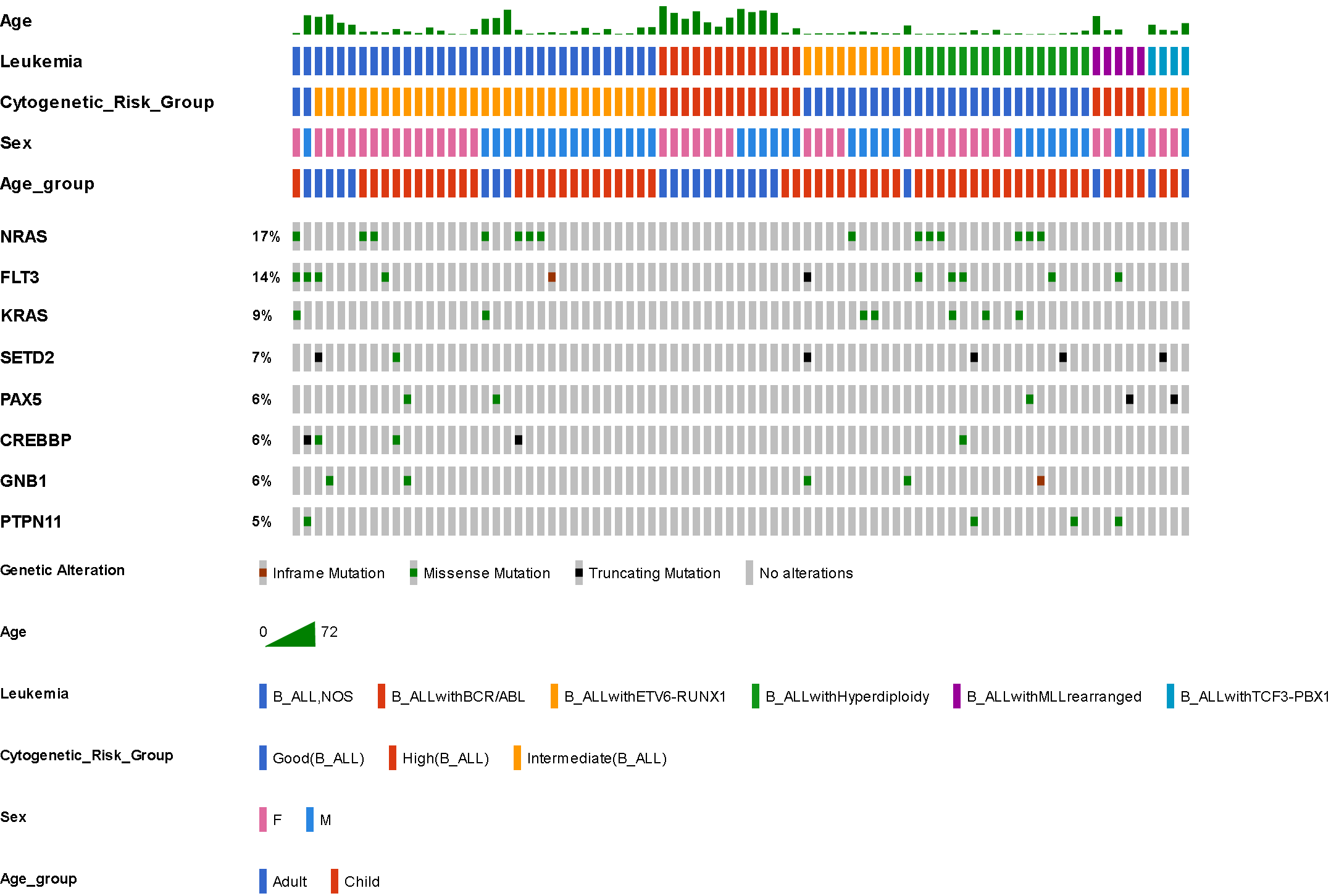


Supplement Fig. S5. Common sequence variants in B-cell acute lymphoblastic leukemia/lymphoma (B-ALL). Only eight most common genes were shown in figure. **Truncating mutations** (nonsense, frameshift deletion, frameshift insertion, splice site); ****i**nframe** (inframe deletion, inframe insertion). Data were analyzed by OncoPrinter (cBioPortal Version 1.14.0, Gao et al.Sci. Signal. 2013 and Cerami et al. Cancer Discov. 2012).


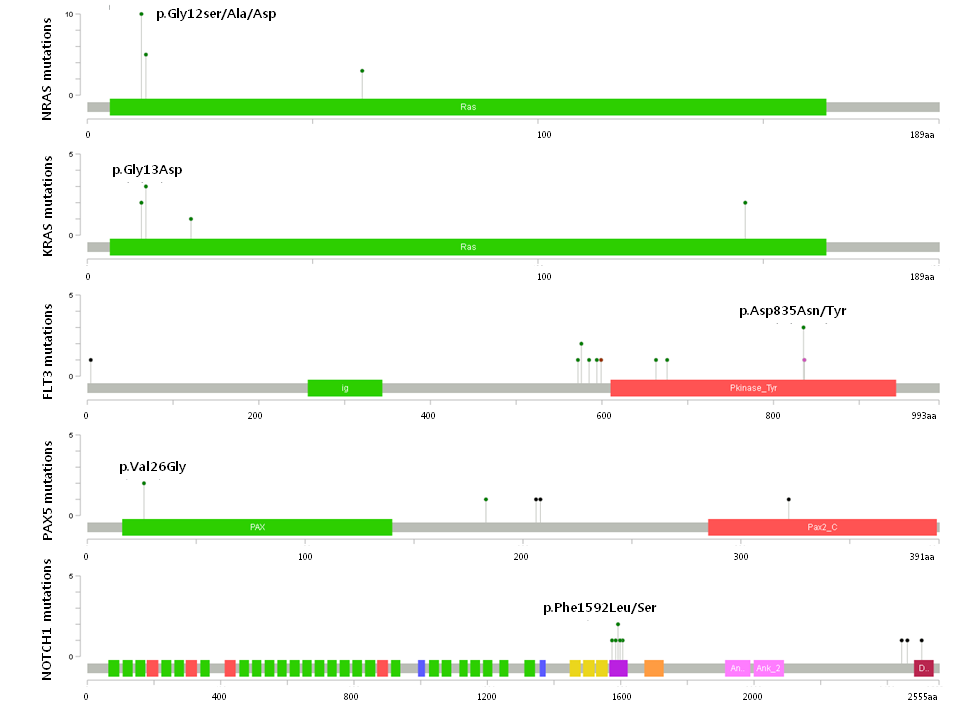


Supplement Fig. S6-1. Lollipop plots of variants (MutationMapper, cBioPortal Version 1.14.0, Gao et al.Sci. Signal. 2013 and Cerami et al. Cancer Discov. 2012). Mutation types and corresponding color codes were as follows: green color, ****Missense****; black color, ****Truncating**** (nonsense, frameshift deletion, frameshift insertion, splice site); brown color, ****Inframe**** (inframe deletion, inframe insertion); pink color, ****Other****


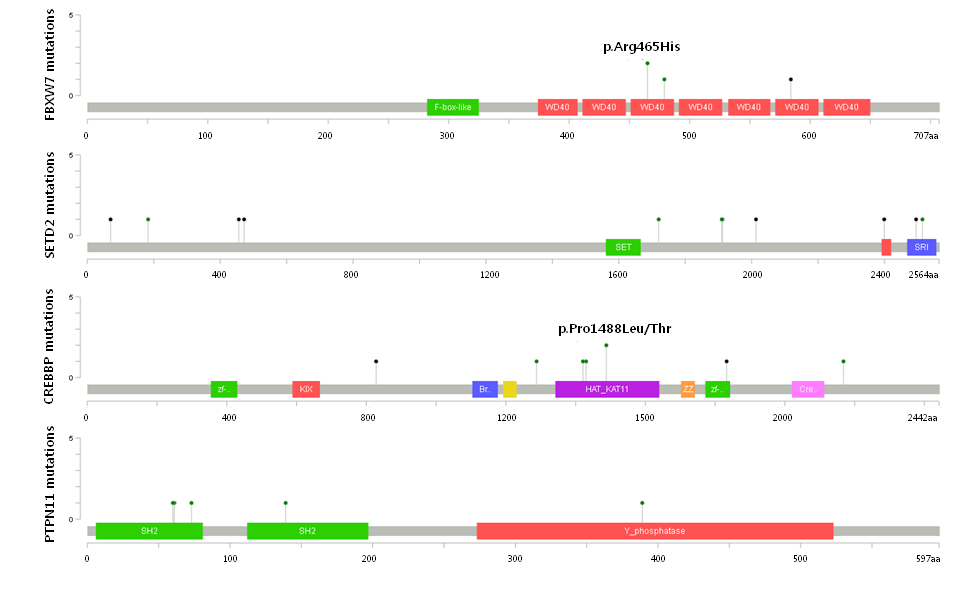


Supplement Fig. S6-2. Lollipop plots of variants (MutationMapper, cBioPortal Version 1.14.0, Gao et al.Sci. Signal. 2013 and Cerami et al. Cancer Discov. 2012). Mutation types and corresponding color codes were as follows: green color, ****Missense****; black color, ****Truncating**** (nonsense, frameshift deletion, frameshift insertion, splice site); brown color, ****Inframe**** (inframe deletion, inframe insertion); pink color, ****Other****.


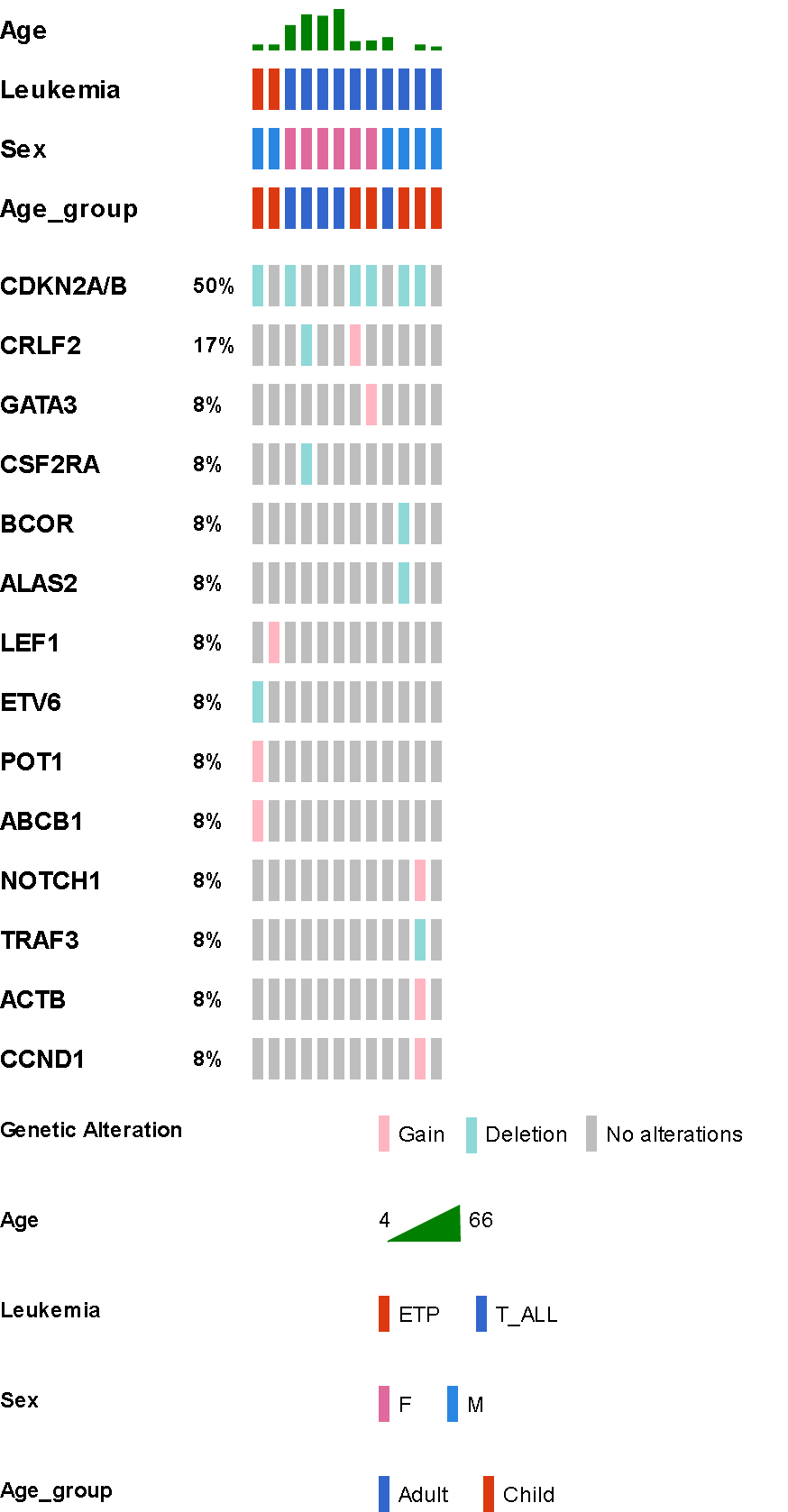


Supplement Fig. S7. Common copy number variants (CNV) in T-cell acute lymphoblastic leukemia/lymphoma (T-ALL). Only fourteen most common genes were shown in figure. Data were analyzed by OncoPrinter (cBioPortal Version 1.14.0, Gao et al.Sci. Signal. 2013 and Cerami et al. Cancer Discov. 2012).


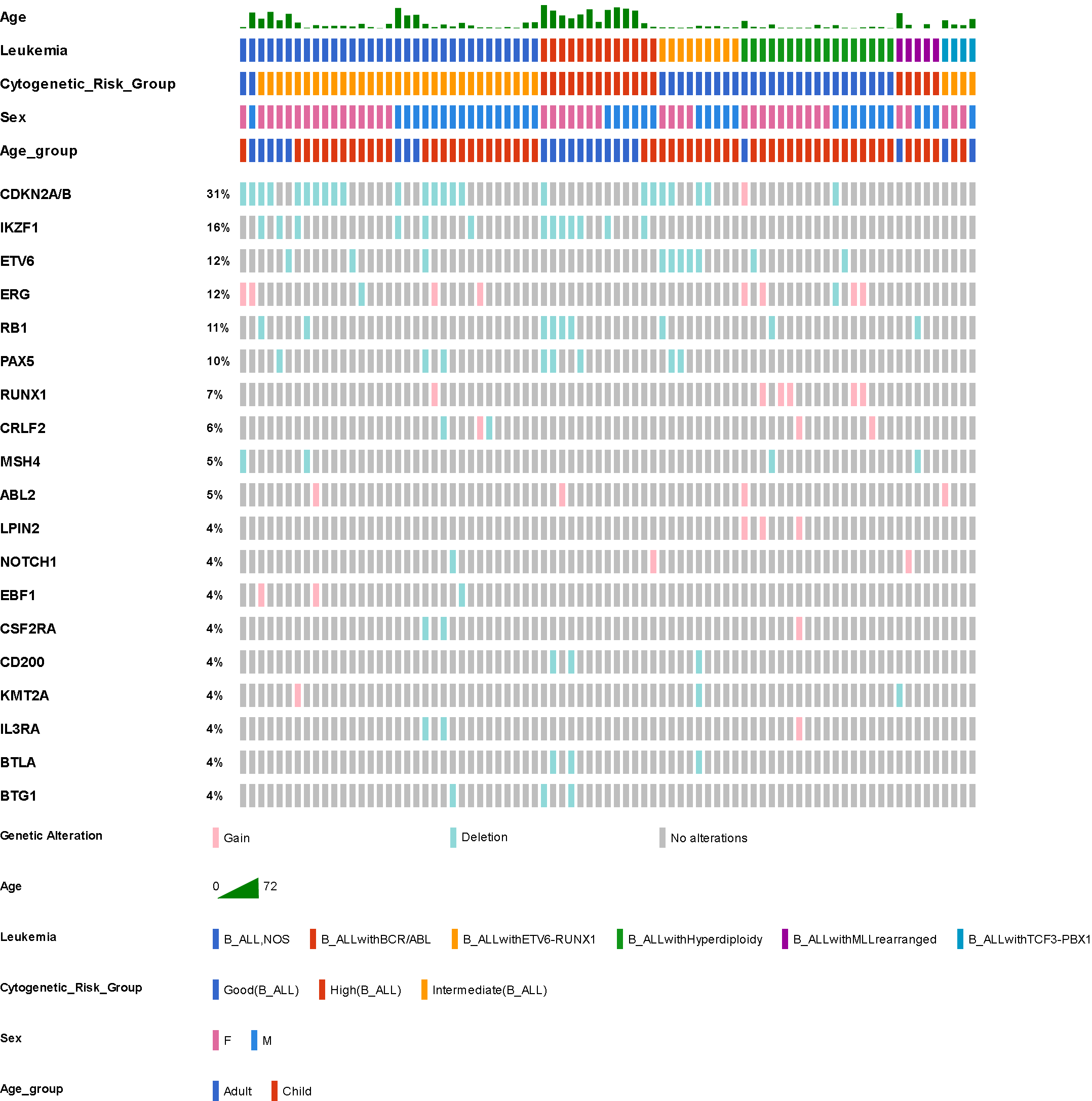


Supplement Fig. S8. Common copy number variants (CNV) in B-cell acute lymphoblastic leukemia/lymphoma (B-ALL). Only nineteen most common genes were shown in figure. Data were analyzed by OncoPrinter (cBioPortal Version 1.14.0, Gao et al.Sci. Signal. 2013 and Cerami et al. Cancer Discov. 2012).


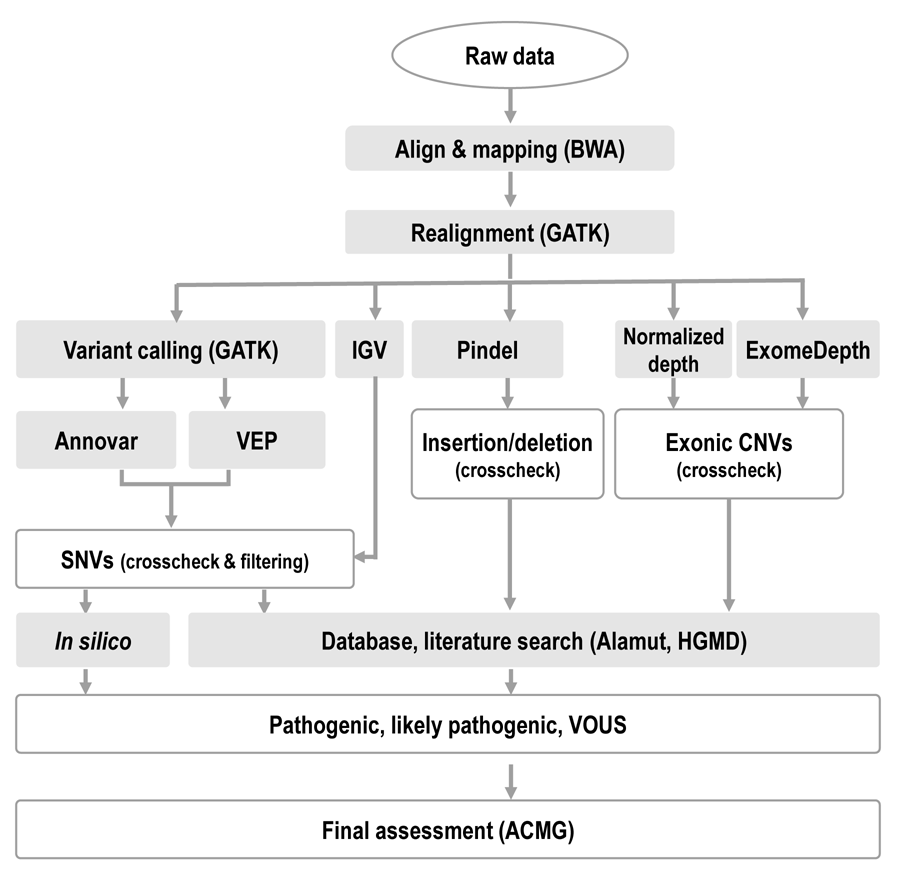


Supplement Fig. S9. Workflow of the next generation sequencing (NGS) data analysis.
